# A Novel Role for the TIR Domain in Association with Pathogen-Derived Elicitors

**DOI:** 10.1371/journal.pbio.0050068

**Published:** 2007-02-13

**Authors:** Tessa M Burch-Smith, Michael Schiff, Jeffrey L Caplan, Jeffrey Tsao, Kirk Czymmek, Savithramma P Dinesh-Kumar

**Affiliations:** 1 Department of Molecular, Cellular, and Developmental Biology, Yale University, New Haven, Connecticut, United States of America; 2 Department of Biological Sciences, Delaware Biotechnology Institute, University of Delaware, Newark, Delaware, United States of America; University of North Carolina, United States of America

## Abstract

Plant innate immunity is mediated by Resistance (R) proteins, which bear a striking resemblance to animal molecules of similar function. Tobacco *N* is a TIR-NB-LRR *R* gene that confers resistance to Tobacco mosaic virus, specifically the p50 helicase domain. An intriguing question is how plant R proteins recognize the presence of pathogen-derived Avirulence (Avr) elicitor proteins. We have used biochemical cell fraction and immunoprecipitation in addition to confocal fluorescence microscopy of living tissue to examine the association between N and p50. Surprisingly, both N and p50 are cytoplasmic and nuclear proteins, and N's nuclear localization is required for its function. We also demonstrate an in planta association between N and p50. Further, we show that N's TIR domain is critical for this association, and indeed, it alone can associate with p50. Our results differ from current models for plant innate immunity that propose detection is mediated solely through the LRR domains of these molecules. The data we present support an intricate process of pathogen elicitor recognition by R proteins involving multiple subcellular compartments and the formation of multiple protein complexes.

## Author Summary

Each year, up to 10% of world agricultural production is lost to pests and diseases caused by a variety of pathogens including bacteria, fungi, nematodes, and viruses. Scientists have understood for nearly a century that plants carry their own immune system that actively engages pathogens and prevents many infections. One aspect of the plant immune system is defined by the gene-for-gene hypothesis: a plant *Resistance (R)* gene encodes a protein that specifically recognizes and protects against one pathogen or strain of a pathogen carrying a corresponding *Avirulence (Avr)* gene. In tobacco and its relatives, the N resistance protein confers resistance to infection by the Tobacco mosaic virus (TMV).

We have used N, and the TMV elicitor, p50, to investigate the mechanism of gene-for-gene resistance. We show that N and p50 associate in the cytoplasm and nucleus of plant cells and that this association is mediated by N's TIR domain, which is structurally similar to animal innate immunity molecules. Our findings provide novel insight into how R proteins recognize pathogen Avr proteins, and should help in long-term efforts to enhance crop yield.

## Abstract 3

How do plant resistance gene recognize the cognate pathogen? These authors dissect the interaction of a resistance gene and a viral pathogen.

## Introduction

Plants, like animals, are able to launch successful defense responses against invading microorganisms. For this purpose, plants have developed a variety of strategies that include molecular, chemical, and physical barriers to infection. One of the most important of these defense systems relies on germline-encoded molecules that can specifically recognize a particular pathogen or strain of a given pathogen. These molecules are encoded by *Resistance (R)* genes, and each R protein typically initiates a defense response in the presence of one pathogen-derived elicitor protein that is termed the Avirulence (Avr) determinant [1]. The genetic relationship between R and Avr proteins was elegantly stated in the gene-for-gene hypothesis [2], and this type of plant defense is now described as the plant innate immunity.

Over the last several years, approximately 40 *R* genes have been cloned [1]. These genes confer resistance to several classes of pathogens, including viruses, bacteria, fungi, oomycetes, insects, and even nematodes. Surprisingly, the protein products of these *R* genes are structurally similar to each other and contain a few, conserved domains. The leucine-rich repeat (LRR) domain is the most common domain among R proteins, and it is also found in animal innate immunity molecules, including Toll from *Drosophila* and Toll-like receptors (TLRs), and nucleotide binding-oligomerization domain proteins (NODs) from mammals [3,4]. Members of the largest class of R proteins possess, in addition to the LRR, a central nucleotide binding site (NB) domain that is similar to the NB of the NODs and the animal cell death effector proteins Apaf1 and CED4 [1,5]. The NB-LRR class of R proteins is further subdivided according to the N-terminal domain of these proteins. Some proteins contain a Toll-interleukin 1 receptor homology region (TIR) domain, whereas others possess a coiled-coil (CC) domain. Like the LRR and NB domains, the TIR domain is found in animal innate immunity proteins, specifically Toll and the TLRs [6].

In recent years, extensive molecular and genetic analyses have been performed in a number of *R-Avr* systems. One interesting aspect of R protein function is its localization. These proteins have been found in a variety of cellular locations, depending on the localization of the eliciting pathogen or its Avr determinant. For example, the tomato Cf proteins, which recognize extra-cellular Cladosporium fulvum Avr proteins, are localized to the plasma membrane [7]. Interestingly, *Arabidopsis* RPM1 and RPS2 are associated with cellular membranes although they do not possess any canonical membrane-targeting domains [8,9]. This subcellular localization is consistent with the membrane localization of their corresponding Avr elicitors, AvrRpm1 and AvrRpt2, respectively [9,10]. Apart from the plasma membrane, R proteins may also be found in the nucleus of plant cells, as is the case with RRS1-R in the presence of its bacterial Avr elicitor, PopP2 [11]. However, many NB-LRR R proteins do not carry recognizable subcellular targeting signatures and so are believed to be cytoplasmic. However, a cytoplasmic localization has only been demonstrated for the *Solanaceae* R protein Bs2 [12] and for barley Mla1 [13]. One caveat to most of these studies is that they involved the generation of biochemical extracts or artificial systems like protoplasts. A more nearly ideal approach for this analysis should use nondisruptive techniques to examine localization in intact, living tissue.

In addition to studies on localization, researchers have also identified some of the host proteins that are involved in R protein activation and signaling downstream of the activation event. Recent work from several groups has attempted to address a central issue in plant innate immunity: how R proteins recognize pathogen-derived Avr proteins and initiate a defense response. Early models of the R-Avr relationship proposed a direct interaction between the host and pathogen proteins based on the gene-for-gene hypothesis. However, a direct interaction has been demonstrated for few R-Avr pairs: [11,14–18]. It should be noted that these interactions have been demonstrated in heterologous systems like yeast and in vitro binding assays. The paucity of detectable R-Avr interactions has led to the hypothesis that other host proteins may facilitate the association of R and Avr proteins and that these accessory host proteins are critical for the activation of the resistance protein. This idea is articulated in the guard hypothesis, which proposes that the Avr protein induces a change in a host protein that is normally recruited by the pathogen via its Avr protein to establish a successful infection, and it is this change that is sensed by the R protein (guard), leading to the activation of the R protein and subsequent defense signaling [19]. This model for R protein activation is supported by evidence from several R-Avr systems. In *Arabidopsis,* both RPM1 and RPS2 and their cognate Avr elicitors interact with a host protein RIN4 [9,20]. RIN4 is modified in the presence of these Avr proteins, and it is believed that this modification is a key step in the activation of the R proteins. Additional support for the guard hypothesis comes from tomato Cf2 and the host protease Rcr3 [21], as well as from *Arabidopsis* RPS5 and PBS1 [22]. Thus, data suggest that some R proteins may indirectly recognize Avr proteins through other host proteins. This mode of activation is in contrast to mammalian TLR function in which TLRs directly recognize pathogen-associated molecular patterns (PAMPs) through their extracellular LRR domains [23]. Interestingly, genetic analyses of plant R proteins have identified a crucial role for the LRR in conferring the specificity of R-Avr systems [14,24],

To date, an in vivo association between an R protein and its corresponding Avr protein has not been demonstrated. This is true even for cases in which a direct interaction has been demonstrated in yeast two-hybrid assays or in vitro. There is no obvious biological explanation for this seeming anomaly. One possibility is that detection has been technically challenging, presumably because R proteins are present at relatively low levels in plant cells [25]. Attempts to increase R protein levels using strong viral promoters like the Cauliflower mosaic virus (CaMV) 35S promoter have failed to yield a wild-type resistance response [26], suggesting that R protein levels are fine-tuned within a cell and that an effective resistance response is dependent on these levels. We have optimized the expression and detection of several R proteins by standard microbiology and molecular biology techniques in an effort to directly address the issues of pathogen recognition and R protein activation.

One of the classic model systems for studying plant-virus interactions involves Tobacco mosaic virus (TMV) infection of Nicotiana tabacum (tobacco) plants. Tobacco and other *Nicotiana* species carrying the *N* gene are resistant to infection by all strains of TMV, except the Ob strain [27]. The *N* gene has been cloned, and it encodes a TIR-NB-LRR R protein [28]. The first visible outcome of infection of *N*-containing plants by TMV is the formation of necrotic lesions at the infection sites [28,29]. These necrotic lesions are part of the stereotypical *R* gene–dependent defense response that is called the hypersensitive response (HR). The helicase domain of the TMV replicase proteins, termed the p50 protein, is necessary and sufficient to elicit an HR in *N*-containing *Nicotiana* plants [30,31]. The HR resulting from *N*–p50 interaction is generally assumed to indicate *N* function [31].

In this work, we have used the N-TMV system to investigate how an R protein recognizes its pathogen-derived elicitor protein. We set out to determine the subcellular localization of N and its Avr elicitor, p50. Using biochemical approaches and fluorescence microscopy, we show that N and p50 are both cytoplasmic and nuclear proteins. We also investigated whether nuclear localization was important for a defense response and found that N's presence in the nucleus was indeed required for its function. However, p50 could elicit *N*-mediated responses even when expressed exclusively in the cytoplasm. Once we determined that N and its elicitor are present in the same subcellular compartments, we then tested the association between N and p50. We show by co-immunoprecipitation and fluorescence microscopy-based assays in intact, living tissue that N and p50 associate in planta. This is the first report of R-Avr association occurring in living tissue undergoing a defense response. Another interesting finding is that the TIR domain of N is critical for this association to occur. These results propose additional functions for the TIR domain in addition to its known role as an adaptor for signaling in animal innate immunity. We propose that the N TIR domain acts as an adaptor between the pathogen Avr protein and the signaling function of the R protein.

## Results

### N and p50 Are Cytoplasmic Proteins

**Figure 1:**
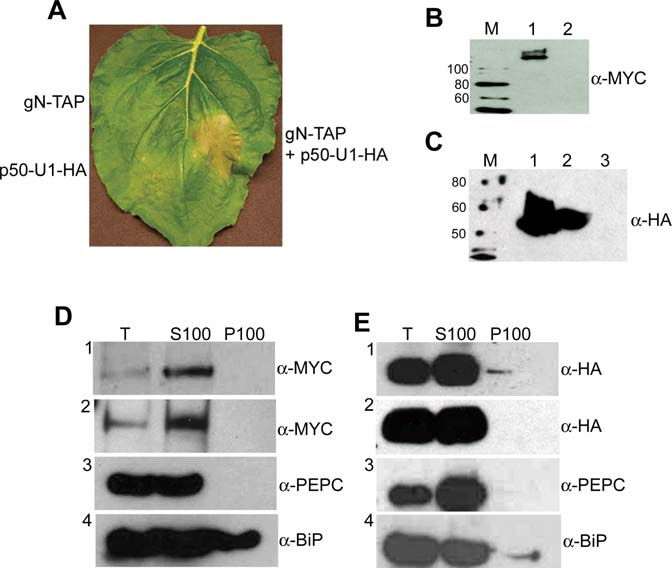
N and p50 Are Found in the Soluble Fraction of Protein Extracts (A) gN-TAP or p50-U1-HA alone do not cause HR cell death in N. benthamiana plants that do not contain *N* (left), whereas co-expression causes death (right). (B) Western blot analysis was used to confirm expression of gN-TAP (lane 1). Lane 2 is an empty vector control. M is the size marker, and protein sizes are shown in kDa. (C) Western blot analysis was used to confirm expression of p50-U1-HA (lane 1) and p50-U1-Ob-HA (lane 2). Lane 3 is an empty vector control. M is the size marker, and protein sizes are shown in kDa. (D) Proteins extracts were centrifuged at 100,000*×g* to produce crude soluble (S100) and membrane (P100) fractions. Fractions were analyzed by Western blot analysis following separation by SDS-PAGE. gN-TAP is found in the soluble fraction in the absence (panel 1) and presence (panel 2) of TMV. PEPC is a cytoplasmic marker, and BiP is found in both the ER and cytoplasm. (E) p50-U1-HA (panel 1) and p50-U1-Ob-HA (panel 2) are both found in the soluble fraction.

In order to localize the N protein, we fused a tandem affinity purification (TAP) tag containing nine copies of the MYC epitope (9xMYC) to the C terminus of N [32]. The full genomic clone of N including its endogenous 5′ and 3′ regulatory sequences and introns was used for this purpose, and the tagged N construct was called gN-TAP. This genomic construct was used to drive expression of N and to facilitate the alternative splicing that is required for N function [33]. To investigate the localization of the TMV elicitor, p50, we used the p50 sequence from the U1 strain of the virus. Two tandem copies of the HA epitope were fused to the C-terminus of p50. Expression of p50-U1-HA was driven by the strong CaMV 35S promoter in an effort to mimic the high levels of viral replicase that accumulate during TMV infection. To determine whether gN-TAP and p50-U1-HA were functional, we co-infiltrated Nicotiana benthamiana leaves with *Agrobacterium* cultures expressing gN-TAP and/or p50-U1-HA. Tissue co-expressing the two proteins exhibited cell death typical of the HR 2 d after co-infiltration (Figure 1A). Tissue expressing either gN-TAP or p50-U1-HA alone did not show the HR cell death response (Figure 1A). The expression of gN-TAP (Figure 1B, lane 1) and p50-U1-HA (Figure 1C, lane 1) was confirmed by Western blot analysis.

The subcellular localization of N and p50 was first examined by cell fractionation. Tissue transiently expressing gN-TAP was collected, and protein extracts were ultra-centrifuged to produce crude soluble (S100) and membrane (P100) fractions. The proteins in the fractions were separated by SDS-PAGE and analyzed by Western blot with anti-MYC antibodies. gN-TAP was found in the S100 soluble fraction of protein extracts in the absence of TMV or p50-U1 (Figure 1D, panel 1), and its localization did not change when co-expressed with its Avr elicitor, p50-U1-HA (unpublished data) or in the presence of TMV itself (Figure 1D, panel 2). Similar analysis for p50-U1-HA showed it to be associated primarily with the S100 fraction (Figure 1E, panel 1). These results suggest that both N and p50-U1 are soluble proteins.

**Figure 2:**
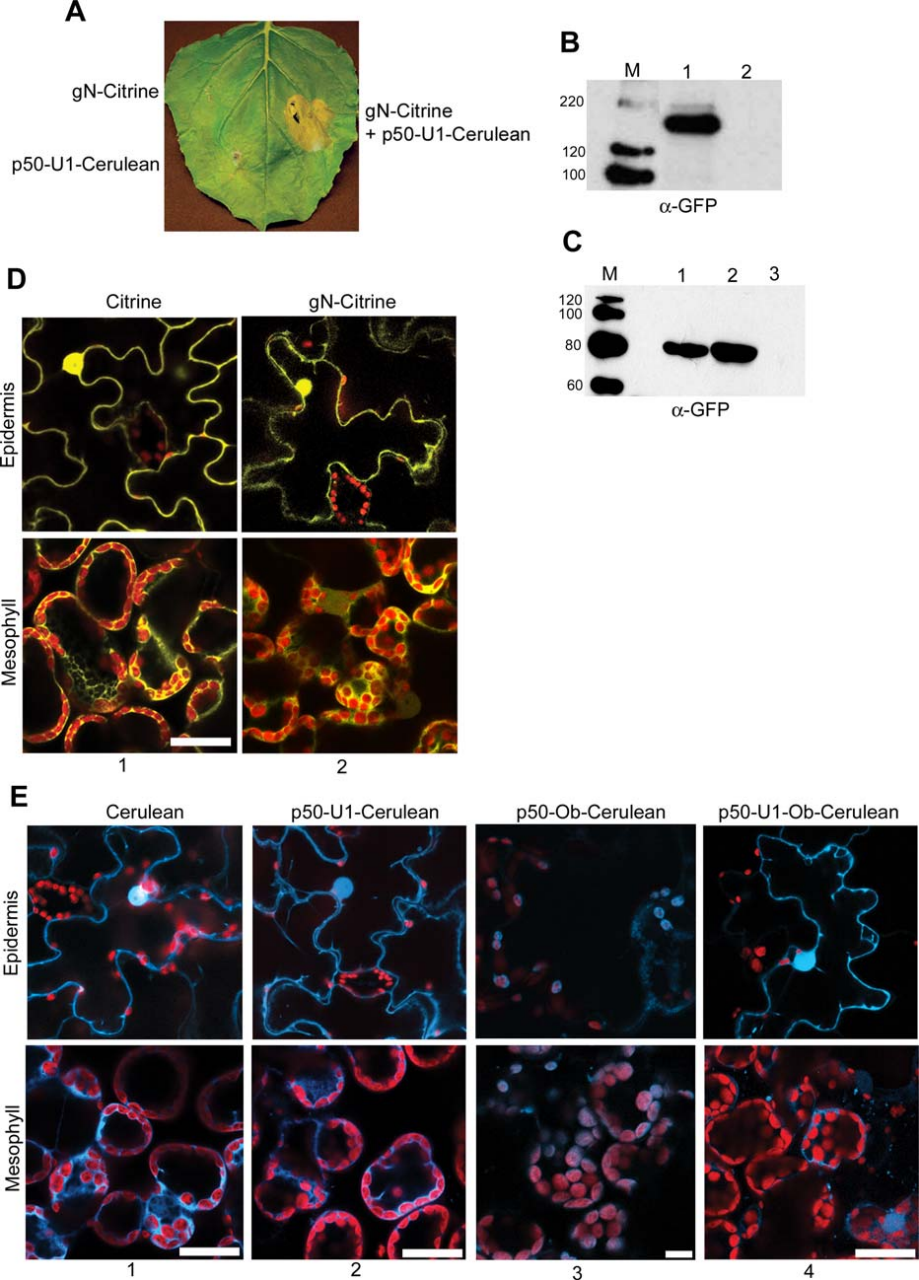
N and p50-U1 Are Cytoplasmic and Nuclear (A) gN-Citrine and p50-U1-Cerulean alone do not cause HR cell death on N. benthamiana plants that do not contain *N* (left), whereas co-expression causes death (right). (B) Expression of gN-Citrine (lane 1) is confirmed by detection with anti-GFP antibodies. Lane 2 is an empty vector control. M is the size marker, and protein sizes are shown in kDa. (C) Expression of p50-U1-Cerulean (lane 1) and p50-U1-Ob-Cerulean (lane 2) is confirmed by Western blot with anti-GFP antibodies. Lane 3 is empty vector control. M is the size marker, and protein sizes are shown in kDa. (D) Localization of gN-Citrine by fluorescence microcopy. gN-Citrine is present in the cytoplasm and nuclei of cells (column 2). Citrine only (column 1) is shown for comparison. Structures in red are chloroplasts. The 514-nm laser line of a 15-mW argon laser and the 543-nm laser line of a 5-mW helium neon laser with appropriate emission filters were used to image Citrine and chloroplast autofluorescence, respectively. Scale bar represents 20 μm. (E) Localization of p50-Cerulean. p50-U1-Cerulean (column 2) is found in the cytoplasm and nuclei of transfected cells. Cerulean alone is shown for comparison (column 1). p50-Ob-Cerulean from a non-eliciting strain of TMV is chloroplastic (column 3), but a p50-U1-Ob-Cerulean chimera shows the same localization as p50-U1-Cerulean (column 4). The 458-nm laser line of a 15-mW argon laser and the 543-nm laser line of a 5-mW helium neon laser with appropriate emission filters were used to image Cerulean and chloroplast autofluorescence, respectively. Scale bars represent 20 μm.

We then used the noninvasive technique of fluorescence microscopy on intact, living leaf tissue as an independent approach to confirm the localization of N and p50-U1. This method avoids the tissue disruption and possible introduction of artifacts that could have occurred during preparation of protein extracts used in our biochemical cell fractionation. The advent of newer forms of fluorescent molecules that give stronger emissions allows the imaging of fusion proteins expressed at fairly low levels [34]. For fluorescence detection, the Citrine variant of enhanced yellow fluorescent protein (EYFP; [35]) was fused to the C-terminus of N. Again, *N* was in its full genomic context including its endogenous 5′ and 3′ regulatory sequences and introns, and Citrine-tagged N was called gN-Citrine. p50-U1 was tagged at its C-terminus with the Cerulean variant of enhanced cyan fluorescent protein (ECFP; [36]) to generate p50-U1-Cerulean. Again, the 35S promoter was used to drive expression of p50-U1-Cerulean. We confirmed that gN-Citrine and p50-U1-Cerulean were functional by assessing their ability to produce HR-associated cell death when co-expressed (Figure 2A). Expression of either construct alone did not result in HR-associated cell death (Figure 2A). gN-Citrine (Figure 2B, lane 1) and p50-U1-Cerulean (Figure 2C, lane 1) were readily detected by Western blot analysis.

Citrine alone or gN-Citrine was transiently expressed in *N. benthamiana* leaves by agroinfiltration. Sections were cut from the infiltrated leaves and observed under the confocal microscope. As expected, Citrine alone, driven by the strong 35S promoter, was localized to the cytoplasm and nuclei of cells (Figure 2D, column 1). Interestingly, gN-Citrine (Figure 2D, column 2) produced a similar pattern of fluorescence to that of Citrine alone (Figure 2D, column 1). These data are consistent with the biochemical analysis of gN-TAP, and show that N is a cytoplasmic protein. However, unexpectedly, gN-Citrine was also detected in the nuclei of most cells examined (Figure 2D, column 2). For p50-U1 localization, a similar pattern of fluorescence was obtained for Cerulean alone (Figure 2E, column 1) and p50-U1-Cerulean (Figure 2E, column 2). Together, these biochemical and cell biological analyses indicate that N and p50-U1 are cytoplasmic and nuclear proteins.

As a control, we were interested in examining a p50 from a TMV strain that could not elicit *N*-mediated defense. The Ob strain of TMV does not elicit *N*-mediated responses at temperatures above 20 °C [27]. As expected, the p50 from the Ob strain of TMV (p50-Ob) does not cause HR-associated cell death when expressed in *N*-containing plants [37]. The amino acid sequences of p50-Ob and p50-U1 are 64% identical and 80% similar [38]. We therefore attempted to characterize p50-Ob. For this purpose, two copies of the HA epitope tag were fused to the C-terminus of p50-Ob to generate p50-Ob-HA. Interestingly, p50-Ob-HA was not detected with antibodies in Western blot analyses (unpublished data). Following the approach used for p50-U1, p50-Ob was tagged with Cerulean to produce p50-Ob-Cerulean. Like p50-Ob-HA, p50-Ob-Cerulean was not detected by Western blot (unpublished data). Surprisingly, we detected low levels of p50-Ob-Cerulean in chloroplasts (Figure 2E, column 3). The fluorescence signal aligns with the stroma of the chloroplasts, and in some cases, stromules (stroma-filled tubules that connect plastids) were identified. It must also be noted that the signal generated by p50-Ob-Cerulean was very weak, even though the 35S promoter was used to drive its expression. It is possible that the failure of p50-Ob to elicit *N*-mediated defense is due to its localization to chloroplasts and exclusion from the cytoplasm or nucleus where N is found.

Analyses of the N-terminus of p50-Ob indicated that it contains a chloroplast localization signal (unpublished data). Therefore, we decided to use a chimeric p50 containing the first 192 amino acids from p50-U1 and the remaining sequence from p50-Ob (p50-U1-Ob) [38]. The p50-U1-Ob chimera fails to elicit *N*-mediated resistance [38]. To investigate the localization pattern of p50-U1-Ob, two tandem copies of the HA epitope tag were fused to the C-terminus of p50-U1-Ob. p50-U1-Ob-HA expression was driven by the 35S promoter, and the protein was detected by Western blot analysis (Figure 1C, lane 2). We examined the localization of the p50-U1-Ob chimera by cell fractionation. p50-U1-Ob-HA was found in the S100 fraction of cell extracts (Figure 1E, panel 2). To determine p50-U1-Ob localization in intact, living leaf tissue, a C-terminal Cerulean tag was attached to this fusion to generate p50-U1-Ob-Cerulean, and it was transiently expressed in N. benthamiana leaves. Unlike p50-Ob-Cerulean, the p50-U1-Ob-Cerulean chimera was detectable by Western blot analysis (Figure 2C, lane 2). p50-U1-Ob-Cerulean was detected in the cytoplasm and nucleus of transfected cells by fluorescence microscopy (Figure 2D, column 4). Thus, although it does not elicit *N*-mediated resistance, the p50-U1-Ob chimera has an identical subcellular localization pattern to p50-U1 that elicits *N*-mediated defense. Moreover, the p50-U1-Ob chimera provides us with a suitable control for our experimental system.

### N's Nuclear Localization Is Required for Defense

**Figure 3:**
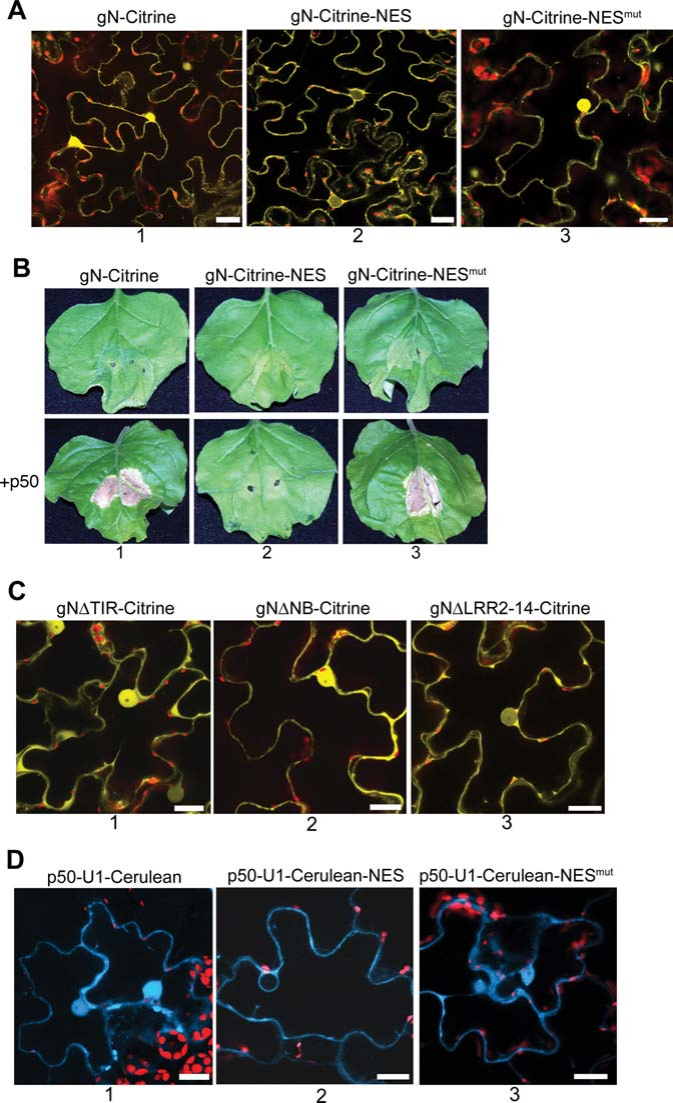
N's Nuclear Localization, but Not p50′s, Is Required for Function (A) Subcellular localization of N with NES. gN-Citrine tagged with a NES is excluded from plant cell nuclei and is found only in the cytoplasm (column 2). gN-Citrine carrying a mutated NES accumulates in the nuclei of plant cells (column 3), and its distribution is similar to that of gN-Citrine (column 1). Citrine fluorescence was imaged with 514-nm laser line of a 15-mW argon laser. Scale bar represents 20 μm. (B) HR assay for function of nuclear N. gN-Citrine expressed alone does not cause HR (column 1, top), but causes HR in the presence of p50-U1 (column 1, bottom). Nuclear-excluded gN-Citrine-NES does not cause HR in the absence (column 2, top) or presence (column 2, bottom) of p50-U1. gN-Citrine-NES^mut^ causes HR only in the presence of p50-U1 (column 3). (C) Defining N's domain required for nuclear localization. NΔTIR-Citrine (column 1), NΔNBS-Citrine (column 2), and NΔLRR2–14-Citrine (column 3) are cytoplasmic and nuclear. There are reduced levels of NΔLRR2–14-Citrine in the nucleus, but the visibility of the nucleolus confirms its presence (column 3). Scale bars represent 20 μm. (D) Subcellular localization of p50-U1 with NES. p50-U1-Ceruelan tagged with a NES is excluded from nuclei and is found only in the cytoplasm (column 2). p50-U1-Ceruelan-NES^mut^ accumulates in nuclei (column 3), and its pattern of distribution is identical to that of p50-U1-Ceruelan (column 1). The 458-nm laser line of a 15-mW argon laser with appropriate emission filters was used to image Cerulean fluorescence. Scale bars represent 20 μm.

Since N's nuclear localization was unexpected, we investigated whether it was important for a defense response. For this, we prevented N's nuclear accumulation by fusing a nuclear export signal (NES) to the C-terminus of gN-Citrine. The NES sequence was derived from the human immunodeficiency virus-1 (HIV-1) Rev protein [39]. As expected, gN-Citrine-NES was excluded from nuclei and found only in the cytoplasm of plant cells when examined by fluorescence microscopy (Figure 3A, column 2). A mutant NES (NES^mut^) in which critical leucine residues have been substituted with alanine, fails to prevent N's nuclear localization (Figure 3A, column 3), and in these instances, gN-Citrines-NES^mut^'s localization is identical to gN-Citrine (Figure 3A, column 1, and Figure 2D, column 2).

We then co-expressed gN-Citrine, gN-Citrine-NES, or gN-Citrine-NES^mut^ and p50-U1-HA and examined whether an HR occurred. As expected, gN-Citrine and p50-U1-HA produced an HR (Figure 3B, column 1). Interestingly, gN-Citrine-NES and p50-U1-HA co-expression did not result in HR (Figure 3B, column 2), whereas the ability to produce an HR was restored in gN-Citrine-NES^mut^ and p50-U1-HA (Figure 3B, column 3). This suggests that N's nuclear localization is required for a defense response.

To identify which domain of N directed its intracellular distribution, we used previously described mutants of N that carry deletions of the TIR, NB, or LRR domains ([26]; see below). None of these mutants produces an HR cell death in the presence of TMV [26], indicating that all three domains are necessary for mounting a successful defense response. Each of these N deletion mutants was tagged at its C-terminus with Citrine for localization by fluorescence microscopy. Again, N mutants were created in their full genomic context including N's endogenous 5′ and 3′ regulatory sequences and introns. Surprisingly, all three N mutants retained their nuclear and cytoplasmic localization (Figure 3C), although lower levels of the LRR deletion mutants appeared to accumulate in nuclei (Figure 3C, column 3). It should be noted that the TIR, NB, and LRR domains do not together constitute the entire N protein, and that there are regions outside these domains that are unaffected in each of the three deletions. Our data suggest that subcellular distribution of N is determined by amino acid sequences outside of the TIR, NB, and LRR domains, because the distribution of the deletion mutants is similar to that of gN-Citrine.

Similarly, we wanted to determine whether the nuclear localization of p50 was necessary for a defense response. Using the same strategy we had employed to investigate the function of nuclear N, we attached a C-terminal NES to p50-U1-Cerulean to determine its intra-cellular distribution and examine whether an HR still occurred in the absence of nuclear p50-U1. As expected, the NES prevented the nuclear accumulation of p50-U1-Cerulean-NES (Figure 3D, column 2), and the fusion protein was able to enter the nucleus when the NES was mutated (Figure 3D, column 3). When p50-U1-Cerulean, p50-U1-Cerulean-NES, or p50-U1-Cerulean-NES^mut^ was infiltrated into *N*-containing N. bethamiana plants, HR was observed (unpublished data). These results suggest that the nuclear localization of p50-U1 is not required for recognition by N and a subsequent defense response. Taken together, our data indicate that recognition may occur in the cytoplasm of plant cells, supporting the hypothesis that nuclear N has another function in addition to pathogen recognition.

### N and p50-U1 Co-Immunoprecipitate

**Figure 4:**
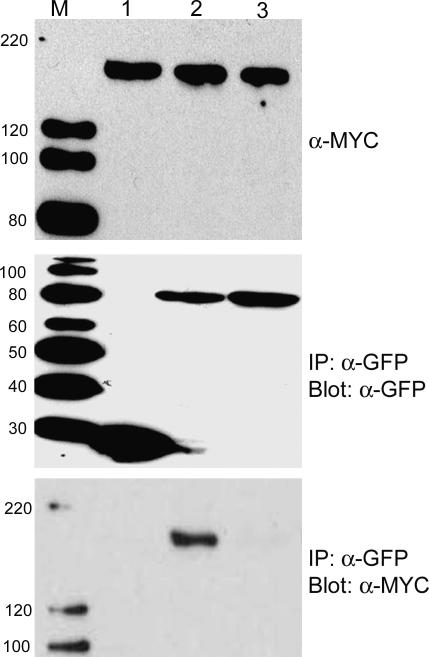
N Co-Immunoprecipitates with p50-U1, but Not with p50-U1-Ob Protein extracts were prepared from N. benthamiana leaves co-expressing gN-TAP (top panel, lanes 1–3) and Cerulean alone (middle panel, lane 1), p50-U1-Cerulean (middle panel, lane 2), or p50-U1-Ob-Cerulean (middle panel, lane 3). Immuno-complexes were pulled down using anti-GFP antibodies. gN-TAP co-immunoprecipitated with p50-U1-Cerulean (bottom panel, lane 2), but not with Cerulean (bottom panel, lane 1) or the non-eliciting p50-U1-Ob-Cerulean (bottom panel, lane 3).

Given that N and p50 were found in the same subcellular compartments, we decided to investigate their association. For this we attempted to co-immunoprecipitate gN-TAP and p50-U1-Cerulean. N. benthamiana plants were infiltrated with a mixture of *Agrobacterium* cultures expressing gN-TAP and p50-U1-Cerulean or Cerulean. We were unsure when these proteins would associate, but we assumed that it would be before HR lesions became visible at 48 h post-infiltration (hpi). Therefore, we allowed sufficient time for *Agrobacterium* to establish a successful infection and T-DNA integration, and then collected samples over a time course from 16 to 48 hpi. Extracts were tumbled with anti–green fluorescent protein (GFP) antibodies to immunoprecipitate p50-U1-Cerulean or Cerulean. Isolated immunocomplexes were analyzed by Western blot, and gN-TAP was detected with anti-MYC antibodies. We found that at 46 hpi, we were able to detect gN-TAP in immunoprecipitated p50-U1-Cerulean complexes (Figure 4, lane 2) but not with those that contained only Cerulean (Figure 4, lane 1). Interestingly, p50-U1-Ob-Cerulean, which does not produce HR when co-expressed with N, was unable to pull down gN-TAP (Figure 4, lane 3). These data demonstrate that N and p50 associate with each other prior to the observation of a visible defense response.

### N and p50 Associate in Intact, Living Tissue

As a second, independent method for assessing the association detected by co-immunoprecipitation, a bimolecular fluorescence complementation (BiFC) assay was used. BiFC, as originally described, splits a fluorescent molecule into two parts that are then individually fused to two proteins whose association is being investigated [40]. If the proteins associate, then the portions of the fluorescent molecule are brought into close proximity with each other, and the fluorescent molecule is reconstituted. It should be noted that BiFC does not require that the associating proteins make direct contact with each other and therefore cannot distinguish between direct and indirect associations [40]. However, BiFC has the distinct advantage of using intact, living tissue to study associations. It does not involve the chemical fixation or physical disruption of tissues and is therefore less likely to produce artifacts.

We used Citrine for the BiFC analysis of N and p50-U1′s association. One tag contained the amino-terminal 155 amino acids (YN_155_), and the other contained the remaining Citrine sequence (YC_155_). As a control to show that the two portions of Citrine could reconstitute fluorescence, published interactions using the 14-3-3 protein, T14-3c, which is known to homodimerize, were repeated [41] (Figure S1A).

**Figure 5:**
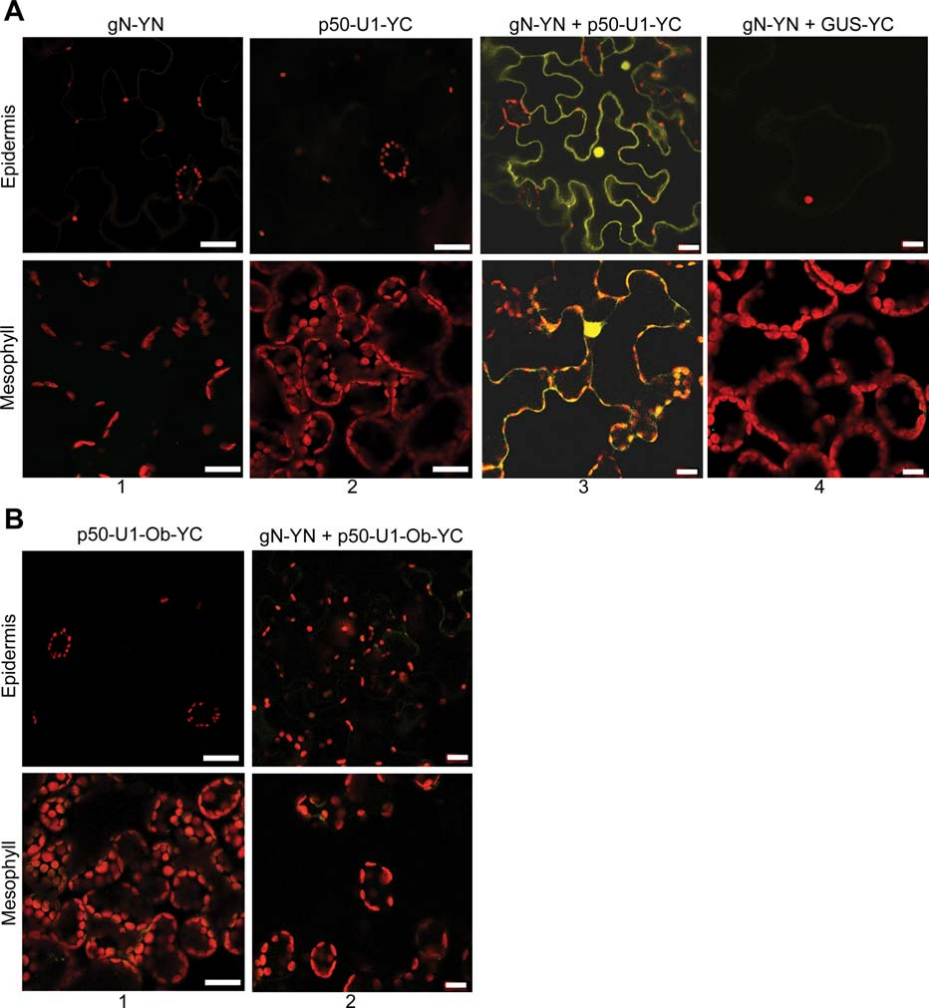
N and p50-U1 Associate In Vivo (A) The BiFC assay was used to demonstrate the ability of N and p50-U1 to associate in living tissue. gN-YN (column 1) alone and p50-U1-YC (column 2) alone do not produce fluorescence in N. benthamiana tissue. Co-expression of gN-YN and p50-U1-YC produces Citrine fluorescence (column 3), demonstrating a close association between N and p50-U1. GUS-YC is used as control for the specificity of associations involving gN-YN (column 4). Citrine fluorescence was imaged with the 514-nm laser line of a 15-mW argon laser. Scale bar represents 20 μm. (B) p50-U1-Ob-YC expressed alone does not produce fluorescence (column 1). Co-expression of gN-YN and the non-eliciting p50-U1-Ob-YC does not produce Citrine fluorescence (column 2), indicating that they do not associate in vivo. Scale bar represents 20 μm.

N, in its full genomic context (endogenous 5′ and 3′ regulatory sequences and introns), was then tagged with YN_155_ to produce gN-YN. p50-U1 was tagged with YC_155_ to give p50-U1-YC, and its expression was driven by the 35S promoter. The tags did not interfere with the activity of N and p50-U1, and they were able to produce HR cell death when transiently co-expressed in N. benthamiana leaves (unpublished data). Samples were collected from plants expressing gN-YC and/or p50-U1-YC at 46 hpi, and observed under the confocal microscope. As expected, gN-YC and p50-U1-YC did not produce any fluorescence when expressed individually (Figure 5A, columns 1 and 2). However, when gN-YN and p50-U1-YC were co-expressed, fluorescence was detected in both cytoplasm and nuclei of cells (Figure 5A, column 3). When we co-expressed gN-YN and a widely used reporter gene, β-glucoronidase (GUS), tagged with YC_155_ (GUS-YC), we were not able to detect any fluorescence (Figure 5A, column 4). Our GUS-YC fusion protein is functional (Figure S1B and S1C), and as expected, GUS-YC alone did not generate fluorescence (Figure S1D). This indicates that the fluorescence we detected with gN-YN and p50-U1-YC was due to a specific association of these proteins.

We also examined the association between N and the p50-U1-Ob chimera. YC_155_ was fused to p50-U1-Ob to produce p50-U1-Ob-YC, and as expected, it did not produce fluorescence when expressed alone (Figure 5B, column 1). Consistent with our co-immunoprecipitation findings, p50-U1-Ob-YC was not able to complement gN-YN, and fluorescence was not observed when they were co-expressed (Figure 5B, column 2). Thus, the failure of this p50 chimera to elicit *N*-mediated responses may be due to its inability to associate with N. Taken together with the co-immunoprecipitation assays, these BiFC results demonstrate that N and p50 associate in plant cells.

Having determined that N and p50 associate, we wanted to examine whether the interaction was direct or indirect. For this, we transcribed and translated N in vitro and performed a co-immunoprecipitation assay with recombinant (HIS)_6_-p50-HA purified from Escherichia coli. We failed to pull down N with p50 in this direct binding assay (Figure S2). A recent publication has shown that full-length N and p50 interact directly in yeast two-hybrid assays, but interestingly, this interaction was not demonstrated by in vitro pull down [18].

### N's TIR Domain Is Necessary for Association with p50

**Figure 6:**
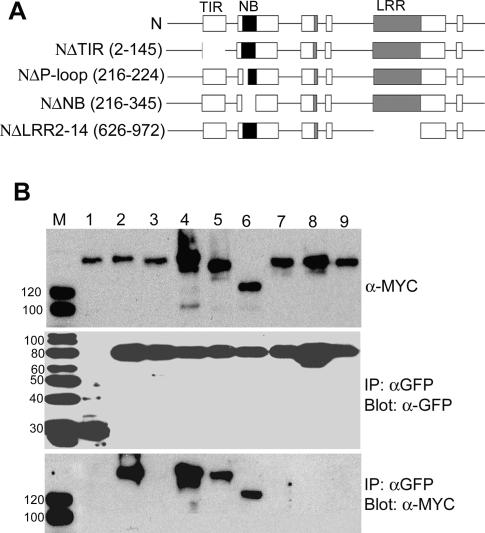
p50-U1 Fails to Associate with N TIR Mutants (A) N and deletion mutants used to determine the domain required for association with p50. Numbers in parentheses are deleted amino acid residues. Not drawn to scale. (B) Co-immunoprecipitation of gN-TAP and N-mutant-TAP proteins with p50-U1-Cerulean. Top panel shows input of N and its mutants, middle panel shows Cerulean-tagged proteins immunoprecipitated with anti-GFP antibodies, and the bottom panel shows the N and N-mutants co-immunoprecipitated. gN-TAP + Cerulean (lane 1); gN-TAP + p50-U1-Cerulean (lane 2); NΔTIR-TAP + p50-U1-Cerulean (lane 3); NΔP-loop-TAP + p50-U1-Cerulean (lane 4); NΔNB-TAP + p50-U1-Cerulean (lane 5); NΔLRR2–14-TAP + p50-U1-Cerulean (lane 6); N(D46H)TAP + p50-U1-Cerulean (lane 7); N(W141S)TAP + p50-U1-Cerulean (lane 8); and gN-TAP +p50-U1-Ob-Cerulean (lane 9). NΔTIR-TAP and the N-TIR point mutants, N(D46H)-TAP and N(W141S)-TAP, are not pulled down by p50-U1-Cerulean (bottom panel, lanes 3, 7, and 8). gN-TAP +p50-U1-Ob-Cerulean (lane 9) or Cerulean (lane 1) are negative controls. Lane M is the size marker, and protein size is in kDa.

Since N and p50 associate in vivo, we wanted to determine which domain of N was responsible for association. For this, we used previously described mutants of N that carry deletions of the TIR, NB, or LRR domain (Figure 6A). To investigate the ability of the mutants to associate with p50-U1, we fused the TAP tag to their C-termini as described for tagging with Citrine. gN-mutant-TAP constructs were co-expressed with p50-U1-Cerulean in N. benthamiana leaves. At 46 hpi, tissue was collected and protein extracts prepared. Anti-GFP antibodies were used to immunoprecipitate p50-U1-Cerulean from extracts, and the precipitate was probed with anti-MYC antibodies after separation by SDS-PAGE. Surprisingly, mutants missing the P-loop of the NB, the entire NB, or the LRR retained the ability to co-immunoprecipitate with p50-U1-Cerulean (Figure 6B, lanes 4, 5, and 6). Unexpectedly, the mutant lacking the TIR domain did not co-immunoprecipitate with p50-U1-Cerulean (Figure 6B, lane 3).

**Figure 7:**
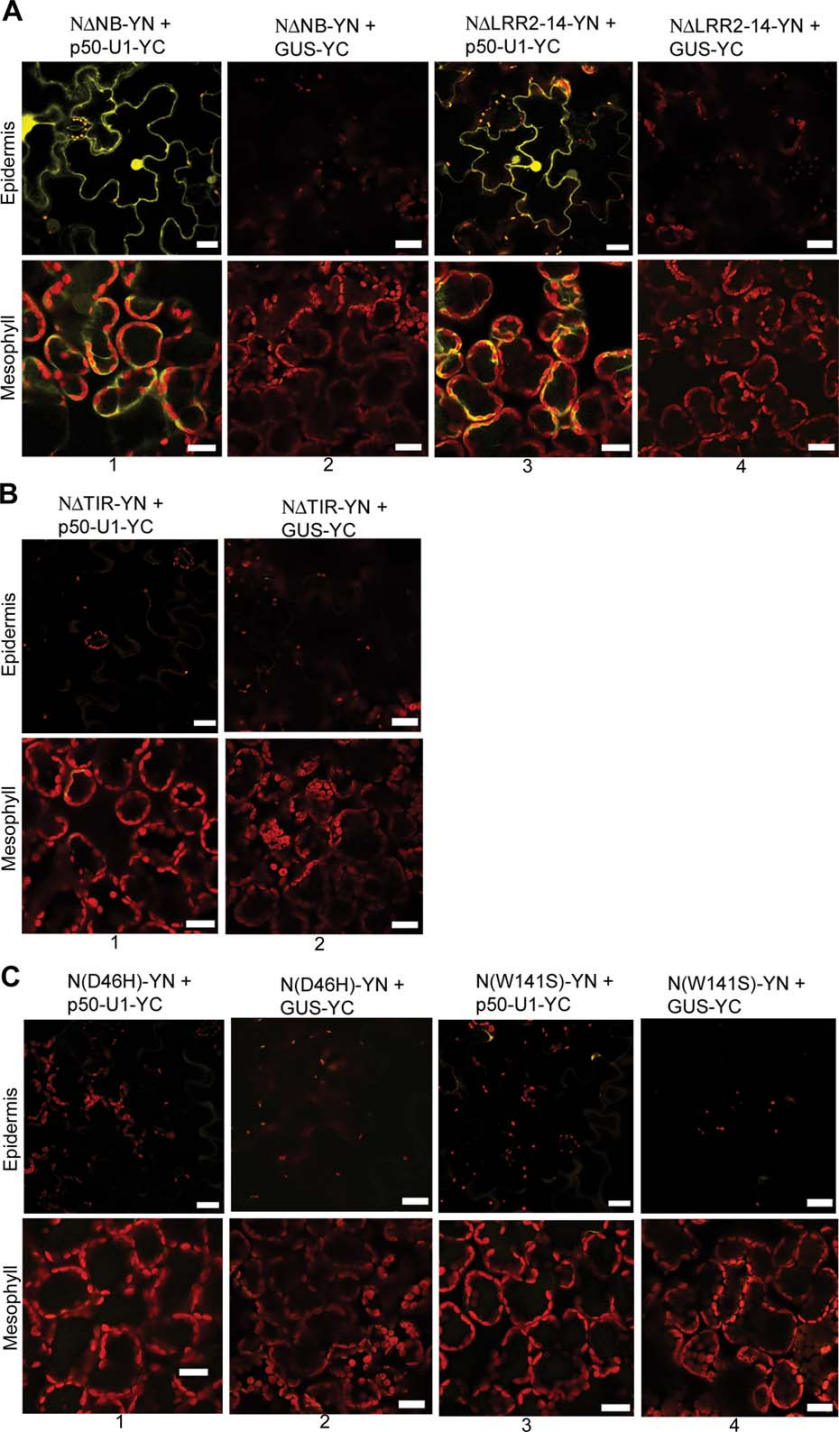
The TIR Domain Is Critical for the Association of N and p50 (A) NΔNB-YN and NΔLRR-YN produce Citrine fluorescence when co-expressed with p50-U1-YC (columns 1 and 3). The specificity of the associations was confirmed by co-expression with GUS-YC (columns 2 and 4). (B) NΔTIR-YN and p50-U1-YC do not exhibit BiFC when co-expressed (column 1). NΔTIR-YN also does not produce fluorescence with GUS-YC (column 2). (C) TIR domain point mutants that disrupt *N*-mediated resistance also do not show BiFC when co-expressed with p50-U1-YC (columns 1 and 3). As expected, they also do not produce fluorescence when co-expressed with GUS-YC (columns 2 and 4). Scale bar represents 20 μm.

These findings were corroborated by the BiFC assay. N mutants lacking the TIR, NB, or LRR domain were tagged with YN_155_, and expression was confirmed by Western blot analysis (unpublished data). Each tagged mutant was then co-expressed with p50-U1-YC or GUS-YC as a control, and tissue was monitored for fluorescence at 46 hpi. The loss of the NB or LRR domain did not disrupt the ability of N and p50-U1 to associate, and fluorescence was detected in those samples (Figure 7A, columns 1 and 3). Again, as in the co-immunoprecipitation assays, the TIR-deletion mutant failed to complement p50-U1, and fluorescence was not observed (Figure 7B, column 1). As expected, none of the mutants gave BiFC with GUS-YC (Figure 7A, columns 2 and 4; Figure 7B, column 2). The TIR domain of N is therefore necessary for association with the Avr elicitor, p50-U1.

It was possible that removing the TIR domain had disturbed the remaining portion of N and this was responsible for the loss of association, not the loss of the TIR domain per se. To examine this possibility, mutants carrying point mutations in their TIR domains were assessed for their ability to associate with p50-U1. Both mutants used, N(D46H) and N(W141S), disrupt *N*-mediated resistance [26]. TAP-tagged point mutants could not be co-immunoprecipitated with p50-U1-Cerulean (Figure 6B, lanes 7 and 8). Similarly, when they were tagged with YN_155_, they failed to complement p50-U1-YC by BiFC (Figure 7C, columns 1 and 3). We had confirmed expression of these mutants by Western blot (unpublished data). These results suggest that the point mutants disturb the function of N by interfering with the ability to associate with its Avr elicitor. Thus, the wild-type TIR domain of N is necessary for association with p50-U1.

### N's TIR Domain Is Necessary and Sufficient for Association with p50

**Figure 8:**
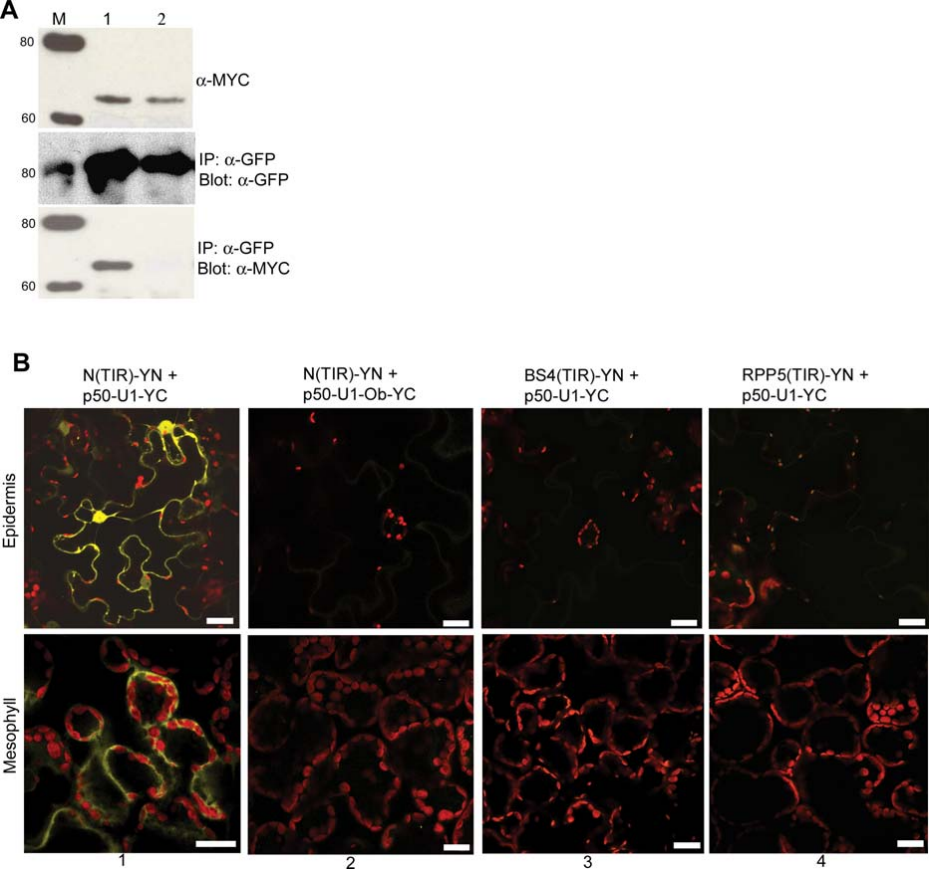
N's TIR Domain Is Sufficient for Association with p50-U1 (A) Co-immunoprecipitation of gN-TIR-TAP and p50-U1-Cerulean. N's TIR domain was expressed under the control of N's endogenous 5′ and 3′ regulatory regions. Extracts from tissue co-expressing N(TIR)-TAP (top panel, lanes 1 and 2) and p50-U1-Cerulean (middle panel, lane 1) or p50-U1-Ob-Cerulean (middle panel, lane 2) were incubated with anti-GFP antibodies. Immunoprecipitated complexes were separated by SDS-PAGE and probed with anti-MYC antibodies. N(TIR)-TAP was pulled down with p50-U1-Cerulean (bottom panel, lane 1), but not with p50-U1-Ob-Cerulean (bottom panel, lane 2). Lane M is the size marker, and protein sizes are shown in kDa. (B) BiFC between N(TIR)-YN and p50-U1-YC. N(TIR)-YN exhibits BiFC with p50-U1-YC (column 1), but not with p50-U1-Ob-YC (column 2). The TIR domains of two related R proteins, BS4 and RPP5, were tested for their ability to associate in vivo with p50-U1-YC. BS4(TIR)-YC and RPP5(TIR)-YC were co-expressed with p50-U1-YC, but were unable to exhibit BiFC (columns 3 and 4, respectively). Scale bar represents 20 μm.

The TIR domain of N was then directly tested for its ability to associate with p50-U1. Using the same strategy applied to full-length N, the TIR domain was TAP-tagged in N's genomic context including its endogenous 5′ and 3′ regulatory sequences to give N(TIR)-TAP. N(TIR)-TAP and p50-U1-Cerulean or p50-U1-Ob-Cerulean were co-transfected into N. benthamiana plants. Tissue was collected 46 hpi, and protein extracts were prepared. Co-immunoprecipitation using anti-GFP antibodies was performed, and the precipitate probed with anti-MYC antibodies. N(TIR)-TAP was found in complexes containing p50-U1-Cerulean (Figure 8A, lane 1), but not in those isolated using p50-U1-Ob-Cerulean (Figure 8A, lane 2).

For the BiFC assay, N-TIR was tagged with YN_155_ to produce N(TIR)-YN, which was then co-expressed with p50-U1-YC or p50-U1-Ob-YC. Consistent with the co-immunoprecipitation results, fluorescence was detected when N(TIR)-YN complemented p50-U1-YC (Figure 8B, column 1). No fluorescence was observed between N(TIR)-YN and p50-U1-Ob-YC (Figure 8B, column 2). As an additional control, we checked for complementation between N(TIR)-YN and GUS-YN, and observed none (Figure S3, column 1). The TIR domain of N is therefore both necessary and sufficient for association with p50-U1.

We then investigated the specificity of the N(TIR)-p50-U1 association. To do this, we chose the TIR domains from two R proteins closely related to N, tomato BS4 and *Arabidopsis* RPP5, and examined their association with p50-U1. N and BS4 share 54% identity and are most similar at the TIR domain [42], whereas the TIR domains of N and RPP5 share 52% identity [43]. The TIR domains of BS4 and RPP5 were each placed under the control of N's 5′ and 3′ endogenous regulatory regions. Thus, the only difference between the N(TIR) and BS4(TIR) and RPP5(TIR) constructs used in our analysis are the coding sequences. We examined the association between BS4(TIR) or RPP5(TIR) and p50-U1 by the BiFC assay. For this, BS4-TIR and RPP5(TIR) were tagged with YN_155_ to produce BS4(TIR)-YN and RPP5(TIR), respectively. The expression of these constructs was confirmed by Western blot (unpublished data). No Citrine fluorescence was observed in tissue co-expressing BS4(TIR)-YN and p50-U1-YC (Figure 8B, column 3) or RPP5(TIR)-YN and p50-U1-YC (Figure 8B, column 4). As expected, BS4(TIR)-YC and RPP5(TIR)-YC expressed with p50-U1-Ob-YC or GUS-YC do not produce fluorescence (Figure S3, columns 2–5). Thus, TIR domains of BS4 and RPP5 do not associate with p50-U1. Thus, the observed association between N-TIR and p50 is specific and depends on the sequence of the TIR domain.

We examined whether the association we observed between N(TIR) and p50-U1 was a direct interaction by pull-down assays, using in vitro transcribed and translated N(TIR) and (HIS)_6_-p50-HA purified from *E. coli.* N(TIR) did not precipitate p50-U1 in this assay (Figure S2), and this is consistent with recent findings [18]. This suggests that the association between N(TIR) and p50-U1 is indirect and may involve other proteins.

## Discussion

**Figure 9:**
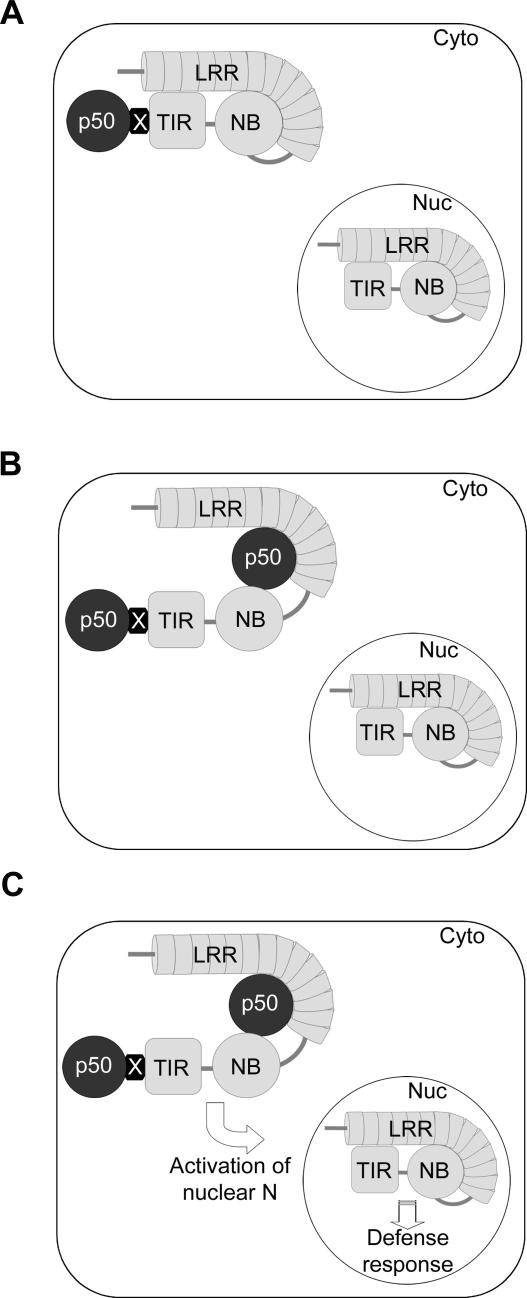
A Model for N Function (A) The first step in recognition. In the cytoplasm (Cyto), p50 (black circle) associates with N through N's TIR domain. The association is bridged by other host factor(s) (X). This may result in conformational changes in N that disrupt interaction of the TIR-NB and LRR and allows oligomerization of N. There is a pool of nuclear N whose function during this time is unknown. Nuc, nucleus. (B) The second step in recognition. p50 then interacts directly with the NB and LRR domains, although it may maintain its association at the TIR domain. (C) The defense response initiation. Following recognition, nuclear N is activated by an unknown mechanism. This leads to signaling that culminates in a defense response.

Here we show that the tobacco R protein N and its cognate Avr determinant from TMV, p50, are cytoplasmic and nuclear proteins. N's nuclear localization is required for a defense response. Further, N and p50 associate in living plant cells as determined by both biochemical and non-destructive microscopic analysis. We have also identified the domain of the R protein that mediates this association. Based on the results of genetic analyses of alleles of *R* genes and several studies of direct interactions by yeast two-hybrid assays, we expected the LRR domain of N to mediate the association with p50. Surprisingly, we found that the TIR domain was not only necessary for association with p50, but also sufficient. We propose a new model for how N may recognize the presence of its elicitor in a plant cell (Figure 9).

We have examined the localization of N and p50 by both biochemistry and fluorescence microscopy. Biochemical assays have been successfully used to localize multiple R proteins [8,9,12,13]. In addition, we utilized confocal fluorescence microscopy to observe the proteins in their native state and subcellular location with minimal disruption to the tissue. For this, we used improved EYFP and ECFP variants, Citrine and Cerulean, respectively, which produce greater fluorescence signal and hence allow easier detection of our fusion proteins [35,36]. Both N and p50 from the U1 strain of TMV are cytoplasmic proteins. This finding was largely expected for N, given that it possesses no obvious subcellular targeting signatures in its sequence and shares sequence similarity to other predicted cytoplasmic R proteins, including BS4 [42] and RPP5 [43].

Surprisingly, in addition to being cytoplasmic, both N and p50-U1 show localization to nuclei. Another R protein, barley MLA1, also shows apparent localization to two subcellular compartments [13]. N's nuclear localization is required for a defense response since preventing its nuclear accumulation of N disrupts the production of an HR. What is the possible significance of N's nuclear localization in mediating a defense response? Interestingly, we had previously identified plant-specific transcription factors as proteins that interact with N [32]. Further, nuclear-localized *Arabidopsis* RRS1 possesses a WRKY DNA-binding domain as a C-terminal extension to its TIR-NB-LRR core structure [11,44]. Taken together, these findings hint at a previously undescribed role for R proteins regulating nuclear events, possibly gene transcription. It will be interesting to determine whether this is indeed the function of N and other nuclear R proteins.

Although p50-U1 is also nuclear, we determined that its nuclear localization was not required for a defense response. In the context of TMV replication, the helicase domain of the viral replicases, p50, is not likely to have access to the nucleus, suggesting that recognition of p50 may occur in the cytoplasm. Thus, our results may indicate that the first phase of defense, recognition of p50, occurs in the cytoplasm while a second, important phase responsible for the actual signaling response occurs in the nucleus. However, we do not yet know how cytoplasmic recognition is communicated to the nucleus.

Despite the availability of cloned *R* and *Avr* gene sequences for the past decade or so, and the demonstration that some R and Avr proteins interact directly with each other in vitro, an association between an R protein and its cognate Avr elicitor had not been previously demonstrated in intact plant tissue. Here we used co-immunoprecipitation and BiFC to show that N and p50 associate in N. benthamiana. It should be noted that co-immunoprecipitation and BiFC do not conclusively distinguish between a direct or indirect association. Therefore, we cannot rule out the possibility that the N-p50 association may be mediated by other host factor(s). Consistent with this idea, host proteins like *Arabidopsis* RIN4 that interact with both an R protein and its corresponding Avr elicitor have been identified [9,20]. Indeed, we failed to demonstrate a direct interaction between N and p50-U1 by in vitro pull-down assay.

Genetic analyses of flax *L* alleles, which encode TIR-NB-LRR R proteins, have pointed to the LRR as being critical for determining the specificity of L-Avr recognition [24]. *L* alleles that differ solely in the sequence of their LRRs confer resistance to different Avr determinants. This suggests that the specificity is derived from the ability of the LRR to associate with, and hence recognize, an Avr protein. This is supported by a recent report of direct L–AvrL interactions in yeast [14]. The LRR domain of the rice R protein Pi-ta has also been shown to interact with its corresponding Avr protein [17] and, interestingly, the NB-LRR region of N interacts with p50 in yeast and in vitro [18]. It is important to note, however, that further analysis of the flax alleles also found that other regions of the L proteins in addition to the LRR domain, particularly the TIR and NB domains, were involved in conferring specificity to L-Avr recognition [45]. Our studies have determined that the TIR domain of N is necessary and sufficient for association with the p50 Avr elicitor (Figure 7A and 7B). Also, the association we observed between N(TIR) and p50 was specific since BS4(TIR) and RPP5(TIR) could not associate with p50 despite their similarity. Our data therefore support a critical role for the TIR domain in mediating the *R–Avr* interaction. Interestingly, results from other R proteins support a possible role for domains other than the LRR in association with pathogen-derived elicitors. For example, *Arabidopsis* RPM1 interacts with the host protein RIN4 through its amino-terminus [20]; RIN4 in turn also interacts with RPM1′s Avr elicitor AvrRpm1. Further, tomato Pto, a kinase that acts as an R protein, interacts with the N-terminus of Prf, a CC-NB-LRR protein required for Pto function [46]. Pto and Prf act closely to regulate not only recognition of pathogen elicitor molecules, but also subsequent defense signaling, and their coordination function depends on their interaction in the plant cell [46]. Thus, it appears that multiple regions of R proteins are involved in interacting with pathogen-derived elicitor molecules, suggesting a complex mode of R protein activation prior to initiating a defense response.

At first glance, our findings appear to contradict two recent papers that investigate the N-p50 association. The first suggests that N and p50 do not associate in plant tissue [47], whereas the other found that N and p50 directly interact in yeast two-hybrid assays, and further, that this interaction occurred through N's NB-LRR region [18]. However, a closer examination reveals that these findings can be assimilated into a coherent model for N's function. In the initial phase of recognition, N and p50 associate through N's TIR domain, most likely through the involvement of other host proteins, because we found this association is indirect (Figure 9A). This is the association that we have detected by co-immunoprecipitation and BiFC in living tissue. The absence of other host proteins from the yeast two-hybrid system may explain why Ueda and co-workers failed to observe the association between TIR and p50 [18]. This N(TIR)–p50 association is possibly the event that leads to the observed oligomerization of N that is proposed to be mediated by N's TIR domain [47]. Next, there is a direct interaction between N and p50 that occurs through N's NB and LRR domains (Figure 9B). This interaction is facilitated through conformational changes of N that result from the disruption of the interaction between the TIR-NB and LRR domains by p50 [18]. This interaction may be weaker than the first, accounting for our failure to observe it in plant tissue. Both interactions likely occur in the cytoplasm because p50 is not needed in the nucleus to initiate a defense response. Subsequently, recognition is communicated to the nucleus, and signaling leading to a defense response follows (Figure 9C). Nuclear-localized N is critical to this process by an as-yet-unknown mechanism, but it may involve changes to the conformation of N, re-distribution of N between the nucleus and cytoplasm, or biochemical modification such as phosphorylation.

We have proposed a complex model for p50 recognition by the N protein that involves different multiple compartments and different regions of N interacting with p50 at different times during the recognition event. Our model also explains the findings from flax (discussed above) that both the LRR and the TIR domains contribute to the specificity of *R–Avr* interactions. Although most of these findings were unexpected, they are consistent with the emerging view that pathogen recognition is a complex process involving players other than the R and elicitor proteins. An intricate recognition system allows for the fine control of the output of this initial event in defense, an important consideration when the outcome for most cells that detect the presence of a pathogen-derived elicitor is death. We expect that our model for N, although differing in the small details, will hold true for other R proteins and their elicitors. It will be interesting to see whether other R proteins are also nuclear-targeted and if so, to determine what function these proteins perform in the nucleus. Also, the investigation of the formation of multiple complexes by different R proteins with their cognate elicitors in addition to high-resolution structures for R proteins will be most useful in explaining these interactions and how they culminate in defense. Finally, it will be exciting to investigate the nuclear–cytoplasmic partitioning of N and other R proteins, and determine the role of these proteins in the nucleus.

## Materials and Methods

### Plasmid constructs.

To generate N-TAP and N deletion mutants-TAP constructs, the TAP tag consisting of 9xMYC-3xHis-3C protease cleavage site-2xIgG binding domain from the vector pYL436 [32] was cloned into the unique SacI site at the 3′ end of the N, NΔTIR, NΔP-loop, NΔNB, NΔLRR2–14, N(D46H), and N(W141)S constructs described in [26]. The Citrine sequence was amplified by polymerase chain reaction (PCR) and was cloned in place of the TAP tag to generate gN-Citrine. The HIV Rev NES sequence (LQLPPLERLTL) and NES mutant sequence (LQAPPAERATL) [39] were included in the downstream PCR primer to amplify Citrine, and cloned into gN to create gN-Citrine-NES and gN-Citrine-NES^mut^. The N-terminal 465 nucleotides of Citrine sequence were amplified by PCR, and cloned into N and N deletion mutants in place of the TAP tag to generate N-YN and N deletion mutants-YN constructs. To generate the TIR domain fused to the TAP and YN_155_ tags, the TIR sequence was PCR amplified and cloned between the NcoI and SacI sites of the gN-TAP and gN-YN constructs. The TIR region of BS4 and RPP5 was PCR amplified from tomato and *Arabidopsis* cDNA, respectively, and cloned between NcoI and SacI sites of gN-TAP. The p50 region of TMV-U1 and Ob replicases was amplified using a primer containing 2xHA sequence, and cloned into pYL400, a T-DNA vector containing the 35S promoter and the NOS terminator cassette. Cerulean and the C-terminal 255 bases of Citrine were cloned into the 3′ end of p50 to generate the p50-Cerulean and p50-YC constructs, respectively. The HIV Rev NES sequence and NES mutant sequence [39] were included in the downstream PCR primer to amplify Cerulean, and cloned into 35S-p50-U1 to create p50-Cerulean-NES and p50-Cerulean-NES^mut^. To produce p50-U1-Ob constructs, p50-U1 sequence (nucleotides 1–576) and p50-Ob sequence (nucleotides 577–1,338) were amplified and inserted in place of p50-U1 sequence in p50-U1-HA, p50-U1-Cerulean, and p50-U1-YC plasmids. PCR-amplified p50-U1-HA was recombined into DEST17 vector (Invitrogen, Carlsbad, California, United States) to generate (HIS)_6_-p50-HA. To generate GUS-YC, the GUS sequence was amplified from pCAMBIA3301 and was inserted in place of p50-U1 in the p50-U1-YC construct. All constructs were confirmed by DNA sequencing.

### Agroinfiltration.

*Agrobacterium* cultures were grown overnight in LB medium containing appropriate antibiotic selections. Cells were pelleted at 3,000 rpm and resuspended in infiltration medium containing 10 mM MgCl_2_, 10 mM 2-morpholinoethanesulfonic acid (MES), and 150 μM acetosyringone, and incubated at room temperature for 2–3 h. Strains containing *N*-derived constructs were infiltrated into N. benthamiana leaves at an optical density (OD_600_) = 1.8, and those containing p50-derived constructs were infiltrated at OD_600_ = 1.0. For co-infiltration, equal volumes of *Agrobacterium* were mixed. Cultures were infiltrated into leaves with a 1-ml needleless syringe. N. benthamiana plants were grown on light carts under 24 h of light, and 4–5-wk-old seedlings were used for all assays.

### Monitoring protein expression levels.

Protein was extracted from ground tissue with buffer containing 150 mM NaCl, 20 mM Tris/HCl, 1 mM EDTA, 1% Triton X-100, 0.1% β-mercaptoethanol, 1 mM PMSF, and complete protease inhibitors (Roche, Indianapolis, Indiana, United States). Protein concentrations were determined by Bradford assay (Bio-Rad, Hercules, California, United States), and equal amounts were loaded onto polyacrylamide gels. Proteins were transferred to PVDF membrane (Millipore, Billerica, Massachusetts, United States) for Western blot analysis. Antibodies used were as follows unless otherwise stated: mouse anti-MYC (Santa Cruz Biotechnology, Santa Cruz, California, United States), rat anti-HA (Roche or Covance, Berkeley, California, United States), mouse anti-GFP (Covance), anti-mouse horseradish peroxidase conjugate (Sigma, St. Louis, Missouri, United States), and anti-rat IgG peroxidase (Roche).

### Localization by ultra-centrifugation.

Samples were ground in liquid nitrogen, and protein was extracted in buffer containing 50 mM HEPES (pH 7.5), 150 mM NaCl, 500 mM sucrose, 10 mM EDTA, 1 mM DTT, 1 mM PMSF, and complete protease inhibitors (Roche). Cell debris was spun down at 10,000*×g* at 4 °C. Extracts were ultra-centrifuged at 100,000*×g* at 4 °C for 1 h. The supernatant or soluble fraction (S100) was collected, and the pellet (P100) was washed with extraction buffer before resuspension in an equal volume of buffer as the S100. Western blot analysis was carried out as described above.

### Co-immunoprecipitation assays.

Plant tissue expressing proteins of interest was collected and ground in liquid nitrogen. Protein was extracted with IP buffer containing 100 mM NaCl, 20 mM Tris/HCl (pH 7.5), 1 mM EDTA, 0.1 % Triton X-100, 10 % glycerol, 1 mM DTT, 2 mM NaF, 1 mM PMSF, and complete protease inhibitors (Roche). Cell debris was pelleted at 20,000*×g,* and extracts were incubated with 50-μl Protein A bead slurry (GE Healthcare, Piscataway, New Jersey, United States) equilibrated in IP buffer. Samples were tumbled at 4 °C for at least 1 h. Protein A beads were spun down at 600*×g* and the supernatant collected. A total of 1-μl rabbit anti-GFP antibodies (Abcam, Cambridge, Massachusetts, United States) were added to each 1-ml sample, and the mixture was tumbled at 4 °C for 2 h. A total of 50 μl Protein A bead slurry equilibrated in IP buffer was added to each sample, and the antibodies were allowed to couple to the beads with rotation for 1 h at 4 °C. Beads were spun down at 600*×g* and the supernatant discarded. Beads were washed three times with IP buffer. A total of 25-μl 4xSDS loading buffer was added to each sample and boiled for 4 min. Immunoblotting was carried out as described previously.

### In vitro pull-down assay.

(HIS)_6_-p50-HA protein was produced in C43(DE3) cells (Lucigen, Middleton, Wisconsin, United States) and affinity purified using nickel-NTA resin (Qiagen, Valencia, California, United States). Approximately 1 μg of purified protein was used to pull down ^35^S-Met-labeled in vitro–translated (TNT; Promega, Madison, Wisconsin, United States) N and N(TIR) as described in [32]. TNT mixture was supplemented with 1.5 mM magnesium chloride and 0.2 mM potassium acetate.

### Fluorescence microscopy.

Live plant imaging was performed on a Zeiss Axiovert 200M light microscope equipped with a LSM 510 NLO confocal microscope (Carl Zeiss, Thornwood, New York, United States) using either a 40× or 63× C-Apochromat water immersion objective lens (numerical aperture [NA] 1.2). Tissue samples were cut from N. benthamiana leaves at approximately 46 hpi and infiltrated with water. The 458-nm and 514-nm laser lines of a 25-mW argon laser (Coherent, Santa Clara, California, United States) and the 543-nm laser line of a 1-mW helium neon laser (LASOS Lasertechnik, Jena, Germany) with appropriate emission filters were used to image Cerulean, Citrine, and chloroplast autofluorescence, respectively. In some instances, 488-nm and 568-nm laser lines of a 15-mW argon:krypton laser (Coherent) were used for Citrine and chloroplast autofluorescence. All images were acquired in fastline switch mode and processed with the Zeiss LSM 510 (Ver. 3.2) channel unmixing algorithm to eliminate crosstalk.

## Supporting Information

Figure S1 Controls Used in BiFC Assay (A) The YN and YC tags are functional when used to test known protein–protein interactions of the 14-3-3 protein, T14-3c (column 1). As expected, by themselves, they do not produce signal (columns 2 and 3). Fluorescence was imaged with the 514-nm laser line of a 15-mW argon laser. Scale bar represents 20 μm.
(B) GUS-YC used in BiFC is functional as shown by blue color from enzymatic assay (left panels), whereas control infiltrated tissue does not show blue color (right panels).
(C) GUS-YC carrying a single HA tag is detected with anti-HA antibodies (lane 1). p50-HA is shown for comparison (lane 2). M is the size marker, and protein size is shown in kDa.
(D) When expressed alone, GUS-YC does not produce fluorescence. Scale bar represents 20 μm.
(7.6 MB TIF)

Figure S2 In Vitro Pull-Down Assay of N and N(TIR) with p50-U1 (A) Western blot analysis of E. coli–purified (HIS)_6_-p50-U1-HA (lanes 1 and 2).
(B) p50 fails to pull-down ^35^S-Met–labeled full-length N (lane 2) and ^35^S-Met–labeled N(TIR) (lane 4) . Five percent of input ^35^S-Met–labeled full-length N (lane 1) and 5% of input ^35^S-Met labeled N(TIR) (lane 2) are shown.
(1.6 MB TIF)

Figure S3 Controls for BS4(TIR) and RPP5(TIR) BiFC Assays N(TIR)-YC (column 1), BS4(TIR)-YC (column 3), and RPP5(TIR)-YC (column 5) do not produce fluorescence when co-expressed with GUS-YC. BS4(TIR)-YC (column 2) and RPP5(TIR)-YC (column 4) also do not exhibit BiFC with p50-U1-Ob-YC.
(5.9 MB TIF)

## Abbreviations

Avr: avirulence
BiFC: bimolecular fluorescence complementation
GFP: green fluorescent protein
GUS: β-glucoronidase
hpi: hours post-infiltration
HR: hypersensitive response
LRR: leucine-rich repeat
NB: nucleotide binding domain shared by Apaf-1, R proteins and CED-4
NES: nuclear export signal
p50: 50-kDa helicase domain of the Tobacco mosaic virus
R: Resistance
TAP: tandem affinity purification
TIR: Toll-interleukin-1 receptor homology region
TLR: Toll-like receptor
TMV: Tobacco mosaic virus
YC: amino acids 155 to 238 of the Citrine variant of yellow fluorescent protein
YN: N-terminal 154 amino acids of the Citrine variant of yellow fluorescent protein
