# Correction: A Novel Role for the TIR Domain in Association with Pathogen-Derived Elicitors

**DOI:** 10.1371/journal.pbio.1002374

**Published:** 2016-01-25

**Authors:** Tessa M Burch-Smith, Michael Schiff, Jeffrey L Caplan, Jeffrey Tsao, Kirk Czymmek, Savithramma P Dinesh-Kumar

There were some errors in the preparation of the middle panel of Fig 4 (which was an inadvertent duplication of Fig 2C), the middle and bottom panel of Fig 6B and the three panels in Fig 8A that were originally published in this article. These errors do not affect the conclusions of the article. The authors wish to correct these figures in order to demonstrate the reproducibility of data presented in Fig 4, Fig 6B and Fig 8A of this article. The authors present results from replicate experiments performed as independent confirmation of the published data (collected in 2007). The corrected data has been verified by the *PLOS Biology* Editors.

The corrected versions of Figs 4, 6 and 8 are included here.

**Fig 4:**
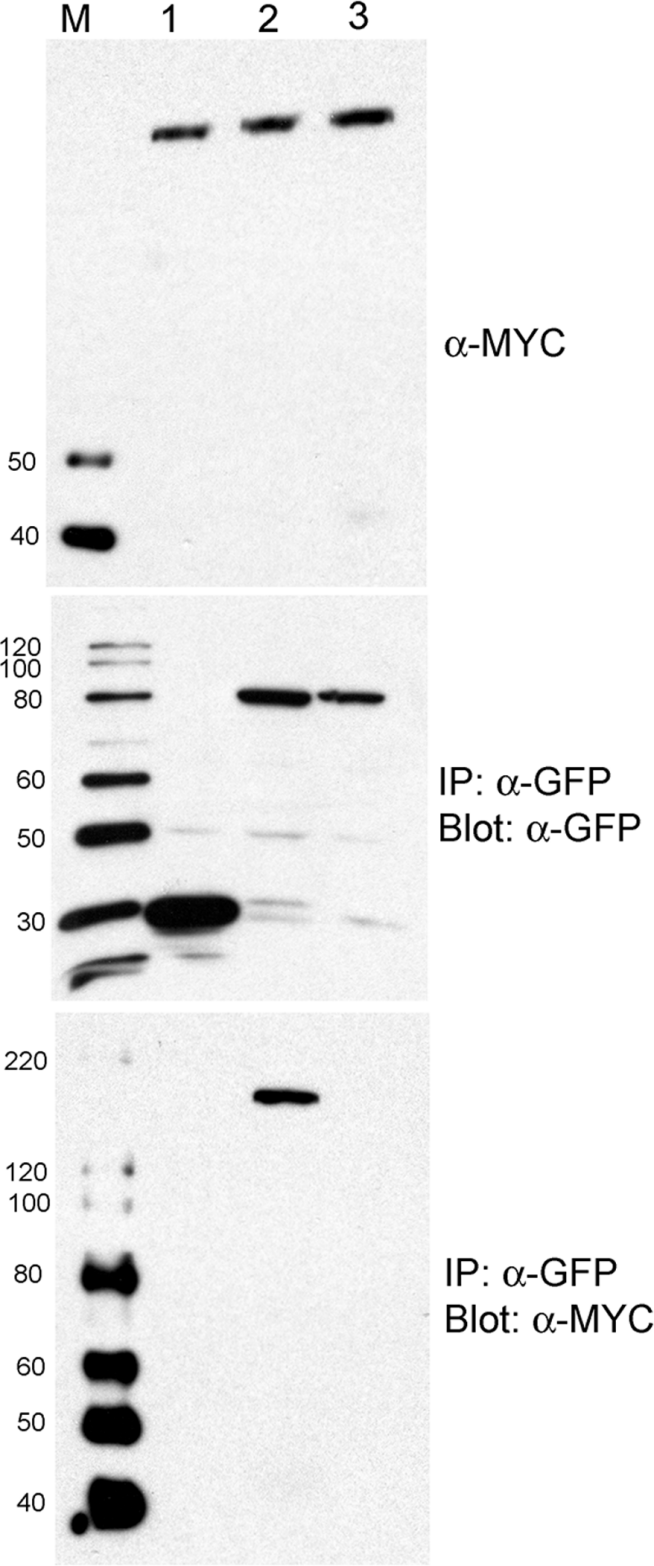
N Co-Immunoprecipitates with p50-U1, but Not with p50-U1-Ob Fig 4. N associates with p50-U1. Total protein extracts from *N*. *benthamiana* leaves transiently coexpressing gN-TAP (top panel, lanes 1–3) and Cerulean alone (middle panel, lane 1), p50-U1-Cerulean (middle panel, lane 2), or p50-U1-Ob-Cerulean (middle panel, lane 3) were pulled down using anti-GFP antibodies. gN-TAP was present in a complex with p50-U1-Cerulean (bottom panel, lane 2), but not with Cerulean (bottom panel, lane 1) or p50-U1-Ob-Cerulean (bottom panel, lane 3).

**Fig 6:**
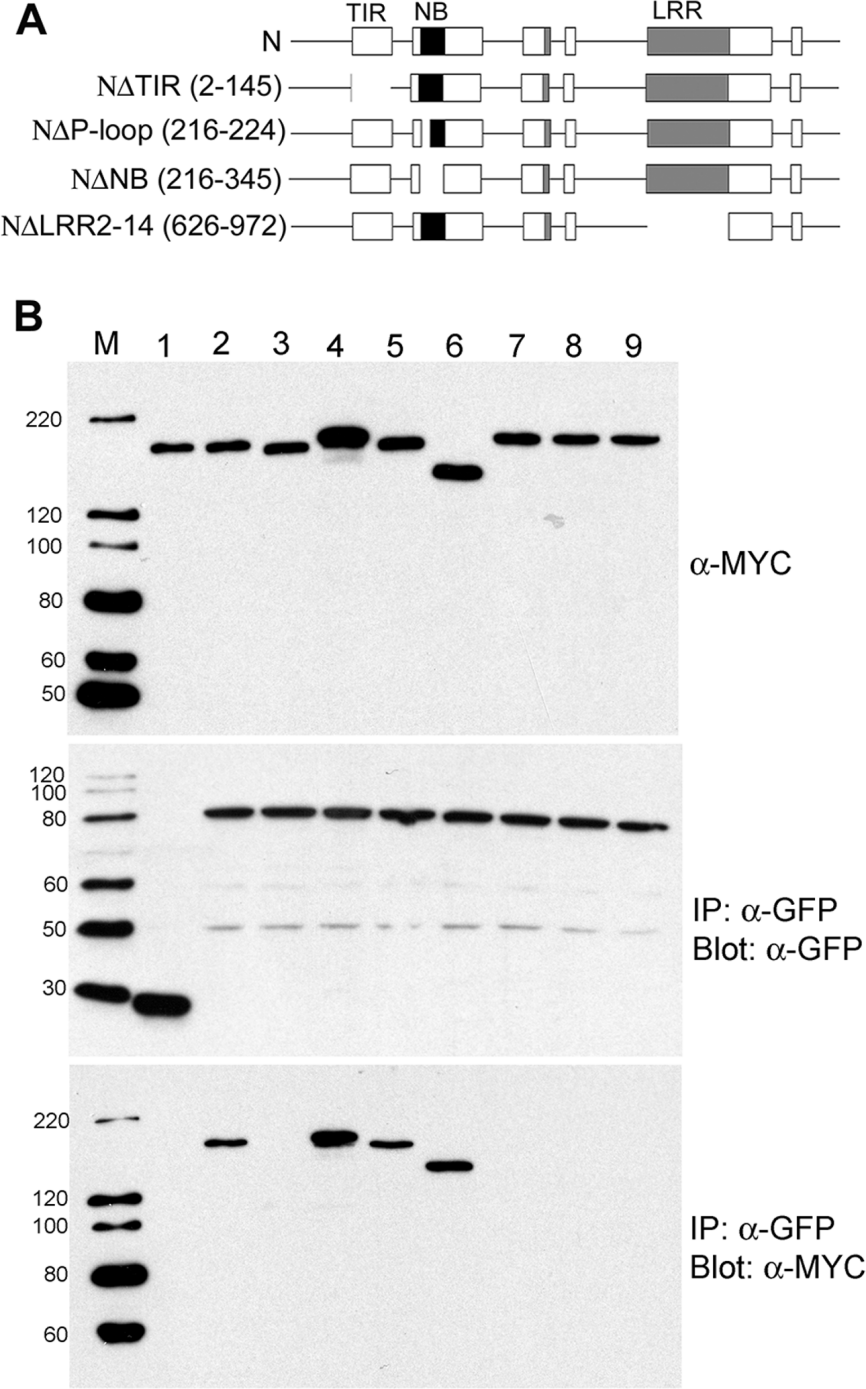
p50-U1 Fails to Associate with N TIR Mutants (A) N and deletion mutants used to determine the domain required for association with p50. Numbers in parentheses are deleted amino acid residues. Not drawn to scale. (B) gN-TAP and gN-mutant-TAP proteins were tested for their association with p50-U1-Cerulean by coimmunoprecipitation. Input of N and its mutants are shown in top panel and Cerulean or Cerulean-tagged proteins are shown in the middle panel. Bottom panel shows coimmunoprecipitation results. gN-TAP + Cerulean (lane 1); gN TAP + p50-U1-Cerulean (lane 2); NΔTIR-TAP + p50-U1-Cerulean (lane 3); NΔP-loop-TAP + p50-U1-Cerulean (lane 4); NΔNB-TAP + p50-U1-Cerulean (lane 5); NΔLRR2–14-TAP + p50-U1-Cerulean (lane 6); N(D46H)TAP + p50-U1-Cerulean (lane 7); N(W141S)TAP + p50-U1-Cerulean (lane 8); and gN-TAP + p50-U1-Ob-Cerulean (lane 9). NΔTIR-TAP and N-TIR point mutants do not coimmunoprecipitate with p50-U1-Cerulean. Lane M is the size marker, and protein size is in kDa.

**Fig 8:**
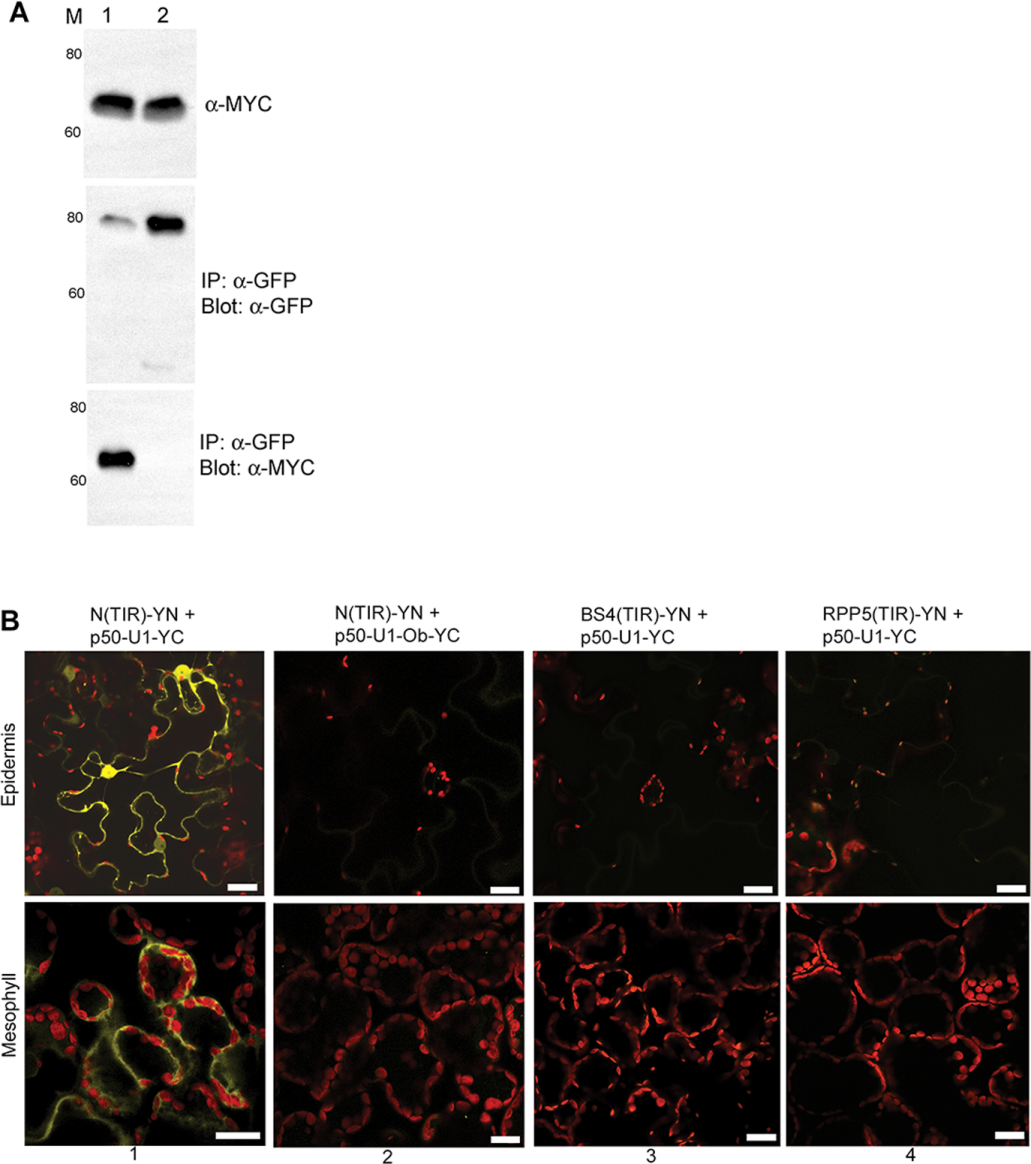
N's TIR Domain Is Sufficient for Association with p50-U1 (A) Co-immunoprecipitation of gN-TIR-TAP and p50-U1-Cerulean. N’s TIR domain was expressed under the control of N’s endogenous 5’ and 3’ regulatory regions. Extracts from tissue co-expressing N(TIR)-TAP (top panel, lanes 1 and 2) and p50-U1-Cerulean (middle panel, lane 1) or p50-U1-Ob-Cerulean (middle panel, lane 2) were pulled down with anti-GFP antibodies. N(TIR)-TAP was pulled down with p50-U1-Cerulean (bottom panel, lane 1), but not with p50-U1-Ob-Cerulean (bottom panel, lane 2). Lane M is the size marker, and protein sizes are shown in kDa. (B) BiFC between N(TIR)-YN and p50-U1-YC. N(TIR)-YN exhibits BiFC with p50-U1-YC (column 1), but not with p50-U1-Ob-YC (column 2). The TIR domains of two related R proteins, BS4 and RPP5, were tested for their ability to associate in vivo with p50-U1-YC. BS4(TIR)-YC and RPP5(TIR)-YC were coexpressed with p50-U1-YC, but were unable to exhibit BiFC (columns 3 and 4, respectively). Scale bar represents 20 μm.
