# Supplementary figures and images for: A Novel Role for the TIR Domain in Association with Pathogen-Derived Elicitors
Source: PLoS Biol. 2007 Feb 13;5(3):e68. doi: 10.1371/journal.pbio.0050068 (PMC1820829; doi:10.1371/journal.pbio.0050068)

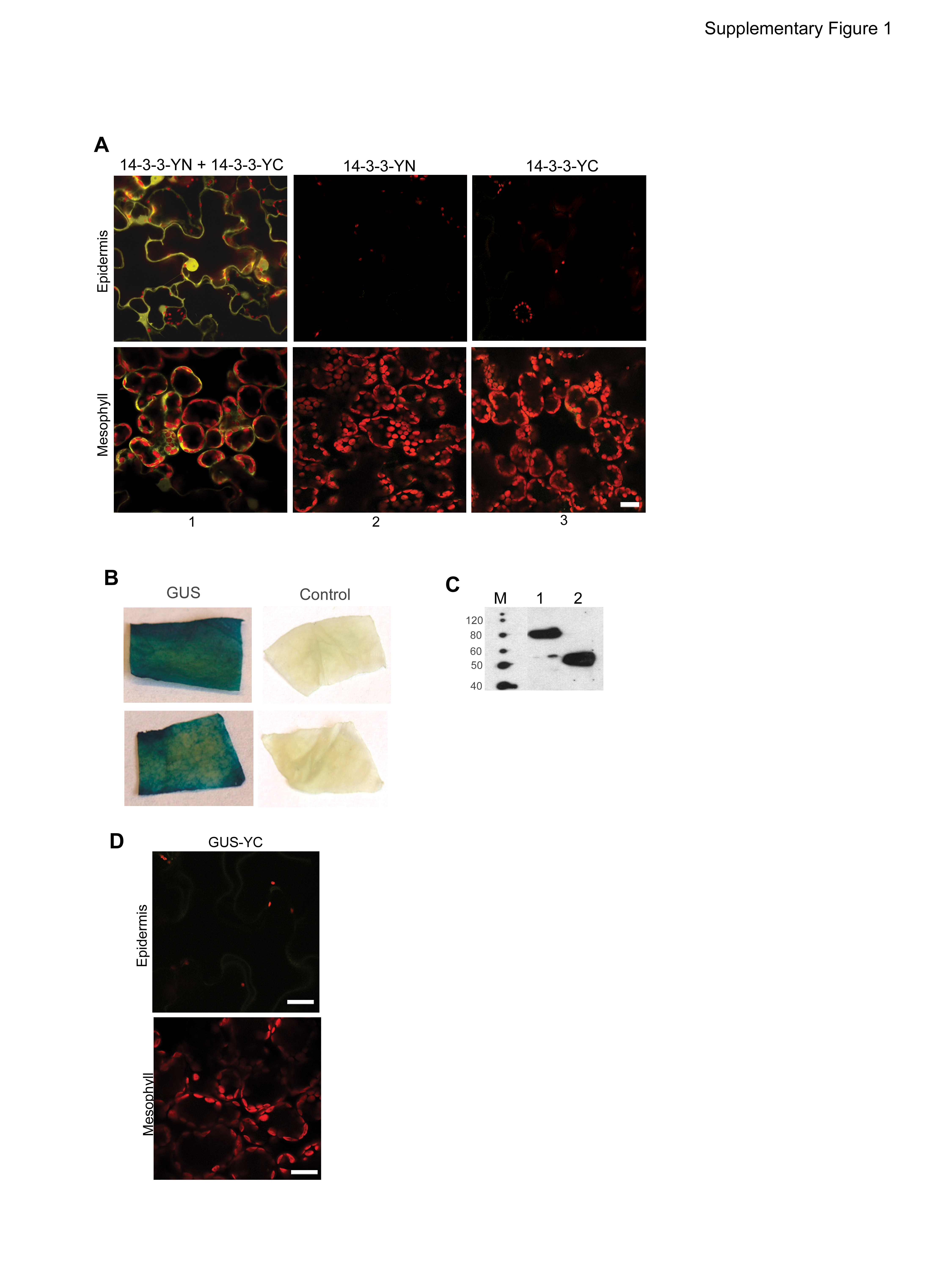

Supplement: Figure S1 — (A) The YN and YC tags are functional when used to test known protein–protein interactions of the 14-3-3 protein, T14-3c (column 1). As expected, by themselves, they do not produce signal (columns 2 and 3). Fluorescence was imaged with the 514-nm laser line of a 15-mW argon laser. Scale bar represents 20 μm. (B) GUS-YC used in BiFC is functional as shown by blue color from enzymatic assay (left panels), whereas control infiltrated tissue does not show blue color (right panels). (C) GUS-YC carrying a single HA tag is detected with anti-HA antibodies (lane 1). p50-HA is shown for comparison (lane 2). M is the size marker, and protein size is shown in kDa. (D) When expressed alone, GUS-YC does not produce fluorescence. Scale bar represents 20 μm. (7.6 MB TIF) [file pbio.0050068.sg001.tif]

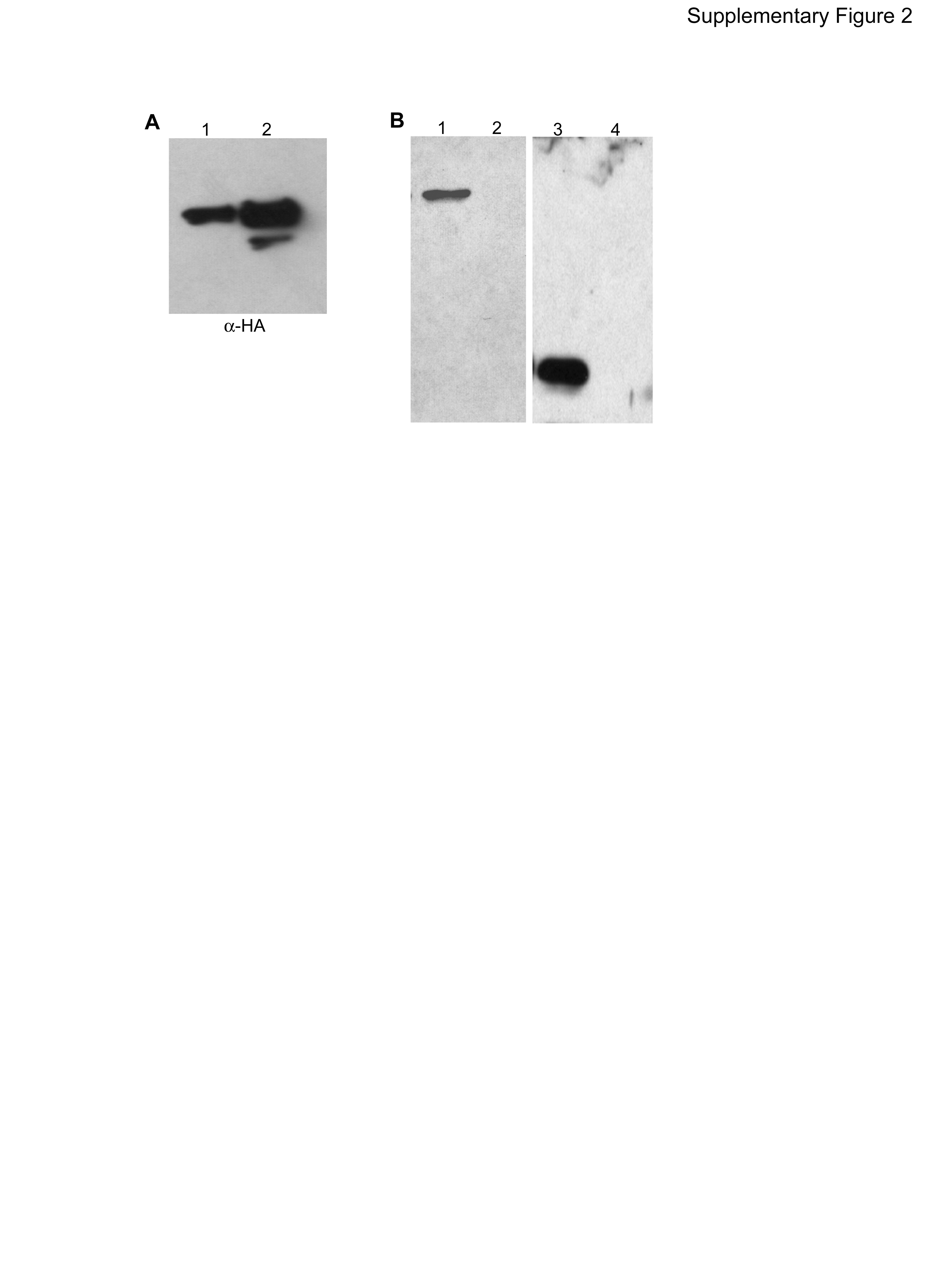

Supplement: Figure S2 — (A) Western blot analysis of E. coli–purified (HIS)6-p50-U1-HA (lanes 1 and 2). (B) p50 fails to pull-down 35S-Met–labeled full-length N (lane 2) and 35S-Met–labeled N(TIR) (lane 4) . Five percent of input 35S-Met–labeled full-length N (lane 1) and 5% of input 35S-Met labeled N(TIR) (lane 2) are shown. (1.6 MB TIF) [file pbio.0050068.sg002.tif]

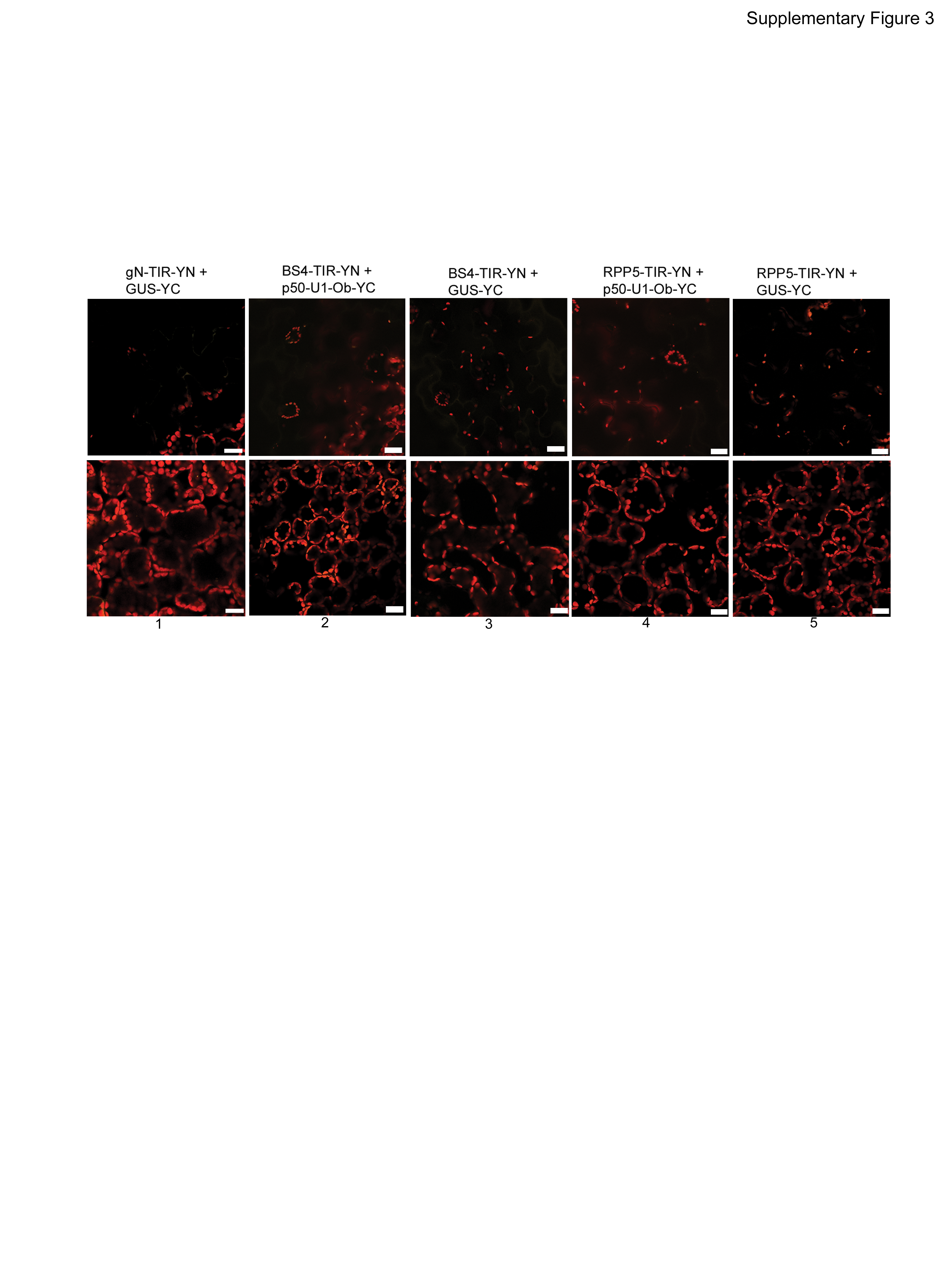

Supplement: Figure S3 — N(TIR)-YC (column 1), BS4(TIR)-YC (column 3), and RPP5(TIR)-YC (column 5) do not produce fluorescence when co-expressed with GUS-YC. BS4(TIR)-YC (column 2) and RPP5(TIR)-YC (column 4) also do not exhibit BiFC with p50-U1-Ob-YC. (5.9 MB TIF) [file pbio.0050068.sg003.tif]
